# *Toxoplasma gondii*, Suicidal Behavior, and Intermediate Phenotypes for Suicidal Behavior

**DOI:** 10.3389/fpsyt.2021.665682

**Published:** 2021-06-11

**Authors:** Teodor T. Postolache, Abhishek Wadhawan, Dan Rujescu, Andrew J. Hoisington, Aline Dagdag, Enrique Baca-Garcia, Christopher A. Lowry, Olaoluwa O. Okusaga, Lisa A. Brenner

**Affiliations:** ^1^Department of Psychiatry, Mood and Anxiety Program, University of Maryland School of Medicine, Baltimore, MD, United States; ^2^Veterans Health Administration, Rocky Mountain Mental Illness Research Education and Clinical Center (MIRECC), Military and Veteran Microbiome: Consortium for Research and Education (MVM-CoRE), Aurora, CO, United States; ^3^Mental Illness Research, Education and Clinical Center (MIRECC), Veterans Integrated Service Network (VISN) 5, VA Capitol Health Care Network, Baltimore, MD, United States; ^4^Department of Psychiatry, Saint Elizabeth's Hospital, Washington, DC, United States; ^5^Department of Psychiatry, Psychotherapy and Psychosomatics, University of Halle, Halle, Germany; ^6^Department of Systems Engineering and Management, Air Force Institute of Technology, Dayton, OH, United States; ^7^Department of Physical Medicine & Rehabilitation, University of Colorado, Anschutz Medical Campus, Aurora, CO, United States; ^8^Department of Psychiatry, Jimenez Diaz Foundation Hospital, Madrid, Spain; ^9^Department of Psychiatry, Madrid Autonomous University, Madrid, Spain; ^10^Department of Psychiatry, Rey Juan Carlos University Hospital, Móstoles, Spain; ^11^Department of Psychiatry, General Hospital of Villalba, Madrid, Spain; ^12^Department of Psychiatry, Infanta Elena University Hospital, Valdemoro, Spain; ^13^Universidad Catolica del Maule, Talca, Chile; ^14^Department of Psychiatry, Centre Hospitalier Universitaire de Nîmes, Nîmes, France; ^15^Department of Integrative Physiology, Center for Neuroscience, Center for Microbial Exploration, University of Colorado Boulder, Boulder, CO, United States; ^16^Menninger Department of Psychiatry and Behavioral Sciences, Baylor College of Medicine, Houston, TX, United States; ^17^Michael E DeBakey VA Medical Center, Houston, TX, United States; ^18^Department of Psychiatry & Neurology, University of Colorado, Anschutz Medical Campus, Aurora, CO, United States

**Keywords:** *Toxoplasma gondii*, suicide, suicidal behavior, suicide attempts, self-directed violence, impulsivity, aggression

## Abstract

Within the general literature on infections and suicidal behavior, studies on *Toxoplasma gondii* (*T. gondii*) occupy a central position. This is related to the parasite's neurotropism, high prevalence of chronic infection, as well as specific and non-specific behavioral alterations in rodents that lead to increased risk taking, which are recapitulated in humans by *T. gondii's* associations with suicidal behavior, as well as trait impulsivity and aggression, mental illness and traffic accidents. This paper is a detailed review of the associations between *T. gondii* serology and suicidal behavior, a field of study that started 15 years ago with our publication of associations between *T. gondii* IgG serology and suicidal behavior in persons with mood disorders. This “legacy” article presents, chronologically, our primary studies in individuals with mood disorders and schizophrenia in Germany, recent attempters in Sweden, and in a large cohort of mothers in Denmark. Then, it reviews findings from all three meta-analyses published to date, confirming our reported associations and overall consistent in effect size [ranging between 39 and 57% elevation of odds of suicide attempt in *T. gondii* immunoglobulin (IgG) positives]. Finally, the article introduces certain links between *T. gondii* and biomarkers previously associated with suicidal behavior (kynurenines, phenylalanine/tyrosine), intermediate phenotypes of suicidal behavior (impulsivity, aggression) and state-dependent suicide risk factors (hopelessness/dysphoria, sleep impairment). In sum, an abundance of evidence supports a positive link between suicide attempts (but not suicidal ideation) and *T. gondii* IgG (but not IgM) seropositivity and serointensity. Trait impulsivity and aggression, endophenotypes of suicidal behavior have also been positively associated with *T. gondii* seropositivity in both the psychiatrically healthy as well as in patients with Intermittent Explosive Disorder. Yet, causality has not been demonstrated. Thus, randomized interventional studies are necessary to advance causal inferences and, if causality is confirmed, to provide hope that an etiological treatment for a distinct subgroup of individuals at an increased risk for suicide could emerge.

## Introduction

### Suicidal Behavior

**Figure 1:**
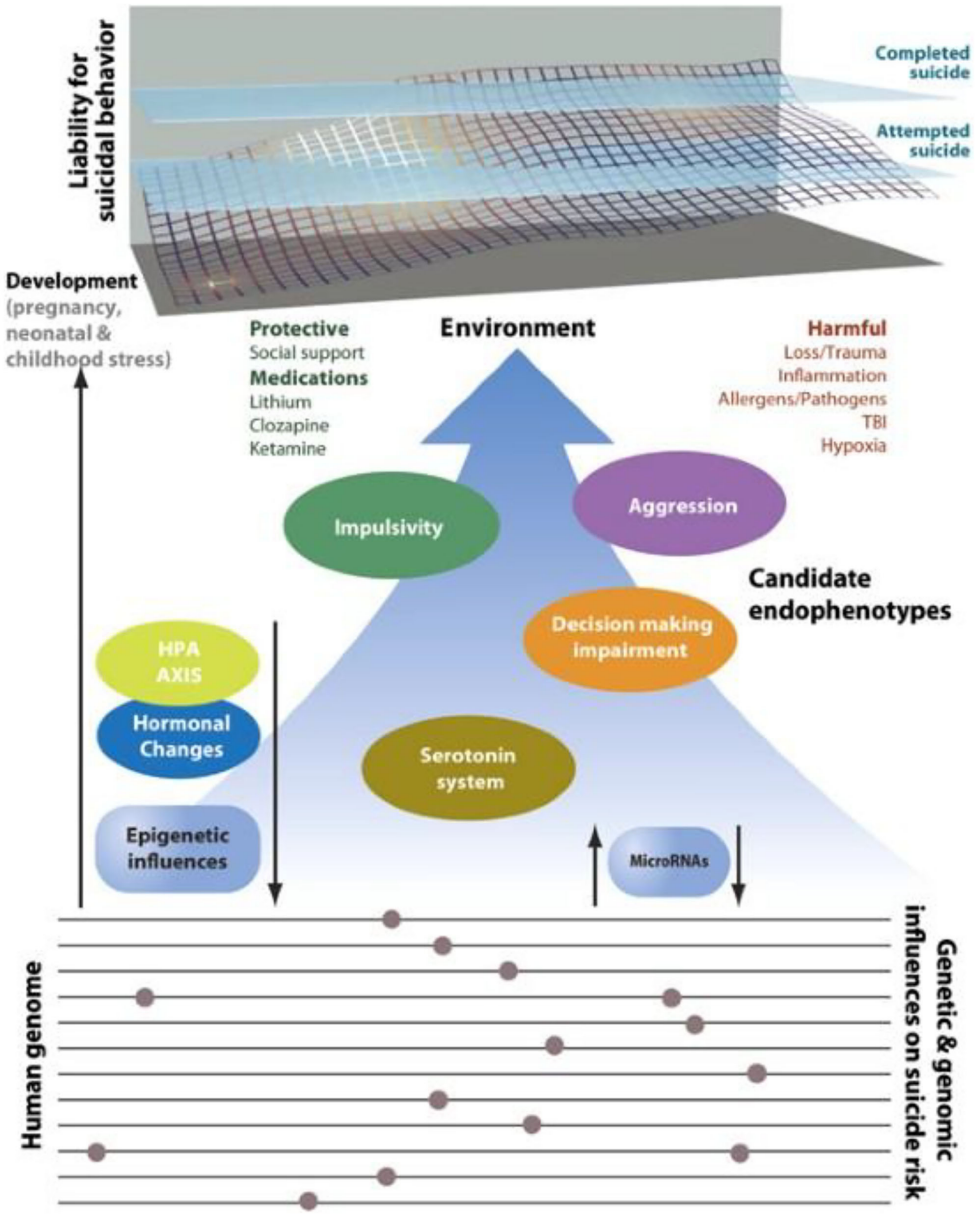
Intermediate phenotypes for suicidal behavior. Model displaying candidate genes, endophenotypes, and environmental risk factors implicated in suicidal behavior that may lend themselves to further study in animal model systems. The upper portion shows the cumulative liability for suicide originating in the dynamic interplay between environmental, genetic, and epigenetic factors. Attempted suicide is a major risk factor but does not always predate suicide as suggested in the figure. Gene loci, genes, candidate endophenotypes and links among these factors remain to be discovered. Many psychosocial stressors are not listed in the figure because of the biological focus. Specific gene loci and genes were not included because of the current limitations in knowledge, and the absence of adequate replication at the time of publication. TBI, traumatic brain injury. *T. gondii* finds its place on the right upper side under “Harmful” environmental factors (Allergens/Pathogens) [Reprinted with permission from (20); Copyright (2020); License link: http://creativecommons.org/licenses/by/4.0/].

Annually, 0.8 million individuals worldwide die by suicide (1). Moreover, every death by suicide is accompanied by 10–20 suicide attempts, leading to an annual number of global suicide attempters of ~10 million (2). Suicidal behavior (including fatal and non-fatal suicidal self-directed violence) is a multi-factorially determined phenomenon (3, 4) in which predispositions and triggers, protective and aggravating factors, availability of means, social and professional supports, as well as deterrents, all interact in a reciprocal interplay that determines short- and long-term risk and prognosis. Interventions geared toward increasing social support, safety (by reducing access to lethal means; e.g., firearm), protective obstacles, hotlines, and education of the public have been recommended as universal (5–7) and selective (3, 6, 8) interventions. Additionally, several explanatory models have been proposed that have usefulness, both in theoretically understanding suicidal behavior among cohorts, and in providing an organized manner by which to characterize specific and dynamic risk factors in individual patients. The models include: (1) the stress-diathesis model (9–12), and; (2) the interpersonal model of suicidal behavior introduced by Joiner, which emphasizes the need for a temporal coexistence of a wish to die (as a result of “thwarted belongingness” and “perceived burdensomeness”) and a capability to engage in suicidal behavior (resulting from habituation to pain and death/dying, often due to repeated exposures to fear-inducing or physically threatening or painful experiences) (13). Several biological factors underlying either vulnerability or triggering of suicide have been proposed and have been summarized and integrated (14). Biological factors that are supported by data include genetic, epigenetic (including microRNAs), endocrine (most commonly implicated are glucocorticoids, gonadal steroids), and neuroimmune factors, sleep and circadian domains (15), neurotransmitters, and brain regions (such as the frontal cortical regions that mediate inhibitory control/impulsivity) (10–12, 16–19). While many of these biomarkers, moderators, or mediators have been previously related directly to suicidal behavior, they are also strongly related to endophenotypes of suicidal behaviors (see Figure 1) (12, 20).

### Contributions to the Field of Neuroimmunology of Suicide Before *Toxoplasma gondii*

Dr. Postolache's group at the University of Maryland School of Medicine has been studying interactions between the biological, chemical and physical environment, and brain and behavior. With multiple national and international collaborators, we had the privilege to contribute with several first-of-their-kind reports. In the neuroimmune domain, we were: (1) the first to identify altered cytokine gene expression (postmortem) in regions of the prefrontal cortex that are implicated in suicidal behavior (21), although in a subsequent work with a different diagnostic composition containing many descendants with substance abuse, we failed to replicate the original findings (22); (2) the first to report an association of immune triggers in spring [such as influenza B and coronaviruses (23) and seasonal pollen peaks] with suicidal behavior (24, 25) that was later replicated by two independent groups (26, 27); and, (3) the first to model the effect of pollen on prefrontal cortex cytokine gene expression, exacerbation of anxiety-like behavior and impairment in social interactions in animal models (28). We were also the first, in collaboration, to report an association between blood kynurenine levels, the initial step of the tryptophan degradation pathway, and a history of suicide attempt in individuals with mood disorders (29). The Postolache group's work on allergens led to identifying, for the first time, mood worsening when individuals with sensitivity to specific aeroallergens [identified by plasma allergen-specific immunoglobulin G (IgG)] were exposed to those specific aeroallergens (30). We also used animal models and found that rodents exhibited aggressive-like behaviors (intermediate phenotypes of suicidal behavior) after a combined stress-allergic challenge [sensitization and exposure to allergens and acute behavioral stress (forced swim test)] (31). Furthermore, Postolache's group reported, pharmacoecologically, lower suicide rates associated with the use of intranasal corticosteroids (medications able to substantially reduce mediators of inflammation in the nasal cavity), relative to new-generation antihistamines (32), which are symptomatically equally effective, but not as effective as intranasal corticosteroids in reducing the local production and potential cerebral translocation of mediators of inflammation. Comorbidity with asthma and its treatment (33), exacerbation of mood disorders (34, 35), or new onset or exacerbation of sleep problems (36) appeared as plausible mediators of emotional and behavioral dysregulation in allergic rhinitis. Furthermore, the nasal-cortical pathway could act as a superhighway (bypassing the blood-brain barrier) for chemical and cellular mediators from the nasal cavity to reach the brain (37). In all, Postolache team's findings strongly suggest that a biological (rather than purely psychological) mediation is in operation, and is responsible for the predictive association between allergic rhinitis and suicidal behavior, and between aeroallergen exposure and suicidal behavior. The team has also published the negative results of its initial failure to replicate (38) its original report (24) of non-violent suicide associations with pollen counts in the United States, and its successful replication in Denmark (25). The convergence of the results of the Postolache team's animal, clinical, and postmortem studies, led to a retreat contemplating and discussing the biological relevance of the uncovered associations, where the idea of including latent infection with *Toxoplasma gondii* (*T. gondii*) in our research portfolio originated (see paragraph A Summary of the Postolache Group's Studies on *T. gondii*, Suicidal Behavior, and Suicide Risk Factors/Intermediate Phenotypes).

### The Postolache Team's Research on *Toxoplasma gondii*, Suicidal Behavior, and Its Intermediate Phenotypes—A Summary

*T. gondii* is an intracellular protozoan parasite that most often results in an asymptomatic or oligosymptomatic infection in approximately one-third of humans worldwide. A relatively lower prevalence (10–15%) has been reported in the United States (US) (39), although a high prevalence has been reported in certain farming communities, such as the Old Order Amish (40). *T. gondii* is zoonotic and can infect any warm-blooded animal. Depending on the degree of immunocompetence of the host and the mechanism of infection, the severity of symptoms can be minimal (in most cases), or severe in (rare cases). If a mother has a primary infection during pregnancy and transmits the infection to her fetus, a potentially devastating congenital infection occurs, with long-term consequences to the offspring. In terms of transmission, felids have been recognized as the definitive hosts of *T. gondii*. The parasite multiplies sexually in the gut of any representative of the felid species, which spreads the oocysts. The ingestion of the parasite within the oocysts by humans and any warm-blooded animal, which play a role of “intermediate hosts,” leads to the spread of the microorganism as tachyzoites (fast-growing forms) from the intestine to other organs, predominantly the muscles and the brain, where it forms cysts containing slow-growing forms (bradyzoites). Further ingestion of these cysts results in closing of the loop of *T. gondii* reproduction cycle in cats, and a secondary spread from the intestine to muscle/brain, with a secondary formation of bradyzoites. In conditions of reduced immune pressure on the parasite, bradyzoites transform into tachyzoites that invade locally, and via circulating immune cells, distally into the host's organs.

### Animal Studies Premising *T. gondii*-Suicidal Behavior Connection

Rodents with chronic (“latent” —i.e., almost undetectable with the naked eye) *T. gondii* infection exhibit significantly altered behavior with associated abnormal neuroendocrine structure and function. The effects of infection can be classified as non-specific (such as increased exploration, enhancing predation by any predator, and thus, leading to formation of cysts in the brain and muscle of these predators) and specific (relating to reduced aversion or even attraction toward cats, the permanent hosts of *T. gondii*). Examples of non-specific effects are lessening of an aversion for open/ less protected spaces and increased novelty-seeking in rodents (41–45). More striking is the reversal of the innate aversion to feline odors in rodents, which allows them to avoid their most common predator (any representative of the cat family), leading to a “fatal attraction” (46–50), is an illustration in mammals of the common behavioral manipulation of the host by the parasite among submammalian organisms. In rodents, the loss of the aversion of predator odors is specific for cats, and not present for other predators that do not play a role of permanent hosts for *T. gondii*. Moreover, this loss of aversion to cat odors is probably an evolutionary phenomenon that increases the parasite's capacity to reproduce, as the ingested meat of infected rodents delivers *T. gondii* to the feline intestinal system, where it undergoes sexual differentiation and sexual reproduction (47, 51).

### Associations Between *T. gondii* and Mental Health

Chronic infection with *T. gondii* has been associated with behavioral, cognitive, psychotic, and affective aberrations in humans (52). Psychiatric disorders, including bipolar disorder (53–57) and schizophrenia (54, 58–65), have been reported to be linked with chronic *T. gondii* infection. Moreover, depression has been reported to be associated with chronic *T. gondii* infection in multiple cohorts, such as pregnant women (66), individuals with mental illness (67) and female Veterans (34). However, the association between *T. gondii* infection and depression has not been replicated in other research studies (53, 68–71). Heterogeneity in the studied samples may have contributed to this discrepancy. Potential risk factors for heterogeneity in the study participants include infection with different *T. gondii* strains, co-morbid substance use disorders, lifestyle variations, different mechanisms of *T. gondii* infection (tissue cyst vs. oocyst), differential associations of individual symptoms of depression with *T. gondii* infection, or variations in genetic vulnerability of the individuals to depression linked with *T. gondii* infection.

### A Summary of the Postolache Group's Studies on *T. gondii*, Suicidal Behavior, and Suicide Risk Factors/Intermediate Phenotypes

Our very simplistic initial thinking was that, if pollen or other aeroallergens are misperceived as invasive pathogens (most likely as parasites because of Th2 cytokine involvement and eosinophil count elevations) and trigger a robust immune defense mechanism that affects brain and behavior, in a sizeable proportion of the population, it is also likely that there are candidate parasites that invade humans with a similar rate as aeroallergens, but do not by themselves cause more harm than the consideration of several alternatives, *T. gondii* seemed, by far, the most likely candidate. *T. gondii* is a small, intracellular parasite, with seropositivity rates (72) that were similar to airborne allergy (73). It is distinctly neurotropic and had already been implicated in mental illness. Being fortunate to have collaborators with the needed expertise, and available samples and data, we first, successfully identified positive associations between *T. gondii*-specific IgG serointensity and history of suicide attempt in individuals with mood disorders (74). This was the first study connecting *T. gondii* and suicidal behavior; up until then the only articles in PubMed connecting suicide with *T. gondii* were on apoptosis (i.e., “suicidal death of cells”). A study in Turkey was the second to find an association between *T. gondii* and suicidal behavior, this time with both IgG serointensity and seropositivity (75). Subsequently, the Postolache group's intent was to test the uncovered association across diagnostic boundaries, as this appeared to be the simplest confirmation that the relationship between *T. gondii* and suicidal behavior is primary, rather than secondary to exacerbation of mental illness. We followed with a positive association between *T. gondii* IgG serointensity and suicidal behavior in younger persons with schizophrenia in Germany (*N* = 1,000) (76), and with a history of suicide attempt in individuals admitted for suicide attempts vs. healthy controls in Sweden, including the scores on a suicide rating scale used to evaluate risk of suicidal behavior in Sweden (77). We then proceeded with a first longitudinal, large, retrospective cohort analysis in Danish mothers, confirming associations between self-directed violence or violent suicide attempts and *T. gondii* IgG seropositivity and stratified titers obtained from neonatal blood spots from neonates. This was the first study in which measurement of *T. gondii* markers occurred prior to the phenotypic behavioral observation and remains the largest study to date (78).

## Meta-Analytic Confirmation and Replication of the Postolache Group's First Reported Association Between *T. gondii* Serology and Suicidal Behavior

We are going to present the three meta-analyses that are currently available. Primary articles included are listed in Table 1. Although these three meta-analyses were performed with certain methodological differences and they included studies that were not completely overlapping, their conclusions are highly convergent and consistent with respect to the direction of the findings and their magnitude, when compared with the Postolache group's initial articles in patients with mood disorders, schizophrenia, acute attempters, and the Danish cohort of mothers.

**Table 1:** Individual studies used in the three meta-analyses with suicide attempts as the outcome measure presented in the article.

**References/Study**	**Included in Sutterland/Amouei/Soleymani Meta-analysis**	**Country**	**Study design**	**Analysis method**	**Test**	**Age (Year ± SD)/(minimum, maximum)**	**Sex (*N*)**	***T. gondii* sero-intensity reported**	**Control population**	**Case population**	**Results: OR (95% CI) Other relevant findings (*n*, % in cases and controls, respectively)^a^**
Arling et al. (74) [First study reporting that suicide attempters had greater mean *T. gondii* IgG titers than non-suicide attempters (*p* = 0.004)]	Yes/Yes/Yes	USA	Case-control	EIA (S)	IgG	P: NA C: NA	P: (F:47, M:34) C: (F:NA, M:NA)	Yes	Mood disorders and healthy	Mood disorders	1.26 (0.56–2.83) Seropositivity: OR 1.62 (95% CI 0.72–3.65) Geometric Mean IgG values (±S.D.), adjusted for age, gender and race: 0.51 (±0.46) v. 0.37 (±0.50)
Yagmur et al. (75)	Yes/Yes/Yes	Turkey	Case-control	ELISA	IgG IgM	P: (24.31 ± 7.57) C: (24.34 ± 8.01)	P: (F:159, M:41) C: (F:155, M:45)	No	Healthy	Psychiatric disorders	1.79 (1.18–2.71) Seropositivity: 82/200 (41%) v. 56/200 (28%) Matched on age, gender, SES, urbanicity and dietary habits
Okusaga et al. (23)	Yes/Yes/Yes	Germany	Cross-sectional	EIA (S)	IgG	P: (38.6 ± 11.1) C: (37.6 ± 11.9)	P: (F:137, M:214) C: (F:213, M:386)	Yes	Schizophrenia	Schizophrenia	1.18 (0.90–1.54) Seropositivity, adjusted for gender, education, PANSS, duration of illness and plate: Adjusted OR (<38 years): 1.57 (1.03–2.38) Adjusted OR (>38 years): 0.79 (0.53–1.19) Serointensity: Adjusted OR (<38 years): 1.23 (1.04–1.47) Adjusted OR (>38 years): 0.90 (0.76–1.01)
Pedersen et al. (78) [Largest sample size and first prospective study]	Yes/Yes/Yes	Denmark	Cohort	EIA	IgG	P: (15, 61) C: (15, 61)	P: (F:1,005) C: (F:44,783)	Yes	Self-directed violence (mothers giving birth to first child)	Self-directed violence (mothers giving birth to first child)	1.28 (1.12–1.47) Seropositivity: SDV: 168/488 (34%) v. 11,949/44,783 (26%) Serointensity: SDV: IgG levels split into 6 categories with rising adjusted RR (adjusted for age, follow-up time, psychiatric contact and history): 0–24 (seronegative) taken as reference, with 25–45 showing RR of 1.08 (95% CI 0.70–1.58) and eventually >84 showing RR of 1.91 (95% CI 1.25–2.79)
Zhang et al. (77)	Yes/Yes/No	Sweden	Cross-sectional	ELISA	IgG	P: (38.4 ± 14.4) C: (39.8 ± 14.2)	P: (F:31, M:23) C: (F:19, M:11)	Yes	Healthy	Psychiatric disorders	2.75 (0.97–7.83) Seropositivity: 22/54 (41%) v. 6/30 (20%) Age-adjusted log-transformed mean IgG titers (±S.D.): 3.0 (±0.1) v. 2.6 (±0.2)
Alvarado-Esquivel et al. (86)	Yes/Yes/Yes	Mexico	Case-control	EIA	IgG IgM	P: (34.01 ± 10.25) C: (38.26 ± 11.62)	P: (F:119, M:37) C: (F:76, M:51)	Yes	Psychiatric disorders	Psychiatric disorders	0.55 (0.20–1.49) Seropositivity: 7/156 (5%) v. 10/127 (8%) High antibody titers (>150 IU/ml): 7/7 (100%) v. 5/10 (50%)
Samojłowicz et al. (87)	Yes/Yes/Yes	Poland	Case-control	IFA	IgG	P: (20, 89) C: (18, 81)	P: (F:5, M:36) C: (F:7, M:79)	No	People who died suddenly due to disease	People who died suddenly due to suicide	1.65 (0.77–3.55) Seropositivity: 26/41 (63%) v. 41/83 (49%)
Alvarado-Esquivel et al. (88)	No/Yes/No	Mexico	Case-control	EIA	IgG IgM	(36.01 ± 12.48)	(F:123, M:26)	Yes	Psychiatric disorders	Psychiatric disorders	0.27 (0.06–1.26) Seropositive: 2/57 (3.5%) v. 13/92 (14.1%) Serointensity: The frequency of high (>150 IU/ml) anti-*T. gondii* IgG levels was lower (but not statistically significant) in patients with suicide attempt (2/57, 3.5%) than in those (11/92, 12%) without suicide attempt (*p* = 0.13).
Fond et al. (89)	Yes/Yes/Yes	France	Cross-sectional	ELISA (S)	IgG IgM	P: (48.1 ± 12.0) C: (42.3 ± 13.7)	P: (F:29, M:25) C: (F:47, M:51)	Yes	Bipolar disorder type I and II	Bipolar disorder type I and II	2.00 (0.86–4.63) Seropositive: 45/54 (83%) v. 69/97 (71%) Serointensity (mean titer ±S.D.): 3.30 (±1.47) v. 2.84 (±1.69)
Fond et al. (89)	Yes/Yes/Yes	France	Cross-sectional	ELISA (S)	IgG IgM	P: (36.2 ± 11.4) C: (35.4 ± 11.5)	P: (F:13, M:30) C: (F:18, M:53)	Yes	Schizophrenia or schizoaffective disorder	Schizophrenia or schizoaffective disorder	1.42 (0.63–3.18) Seropositive: 30/43 (70%) v. 44/61 (72%) Serointensity (mean titer ±S.D.): 2.68 (±1.69) v. 2.53 (±1.81)
Coccaro et al. (117)	No/Yes/No	USA	Cross-sectional	ELISA (S)	IgG	P: (36.1 ± 8.3) C: (31.3 ± 8.7)	P: (F:45, M:64) C: (F:52, M:58)	No	Different states (healthy, psychiatric and Intermittent Explosive Disorder)	Different states (healthy, psychiatric and Intermittent Explosive Disorder)	1.42 (0.59–3.44)
Coryell et al. (90)	Yes/Yes/Yes	USA	Case-control	ELISA	IgG	P: (17.5 ± 1.7) C: (19.0 ± 1.6)	P: (F:13, M:4) C: (F:65, M:26)	No	Adolescents with mood disorders	Adolescents with mood disorders	5.93 (0.78–45.40) Seropositivity: 2/17 (12%) v. 2/91 (2%) Mean IgG titers (±S.D.): Adjusted for age and gender
Sugden et al. (91)	Yes/Yes/Yes	New Zealand	Cohort	EIA	IgG	P: (38) C: (38)	(F:414, M:423)	No	Psychiatric disorders	Psychiatric disorders	2.60 (0.96–7.01) Seropositivity: Suicide attempt since age 32: 8/236 (3.4%) v. 8/601 (1.3%)
Ansari-Lari et al. (92)	Yes/Yes/Yes	Iran	Case-control	ELISA	IgG	P: (43.5 ± 8.1) C: (38.0 ± 11.1)	P: (F:10, M:32) C: (F:17, M:40)	Yes	Schizophrenia	Schizophrenia	1.03 (0.42–2.51) Seropositivity: 8/42 (19%) v. 21/57 (37%) Mean IgG titers (±S.D.): 7.7 (±11.7) v. 12.5 (±13.7)
Samojłowicz et al. (104)	No/Yes/No	Poland	Case-control	ELISA	IgG	P: (18, 89) C: (20, 89)	P: (F:13, M:113) C: (F:25, M:140)	No	Individuals who died as a result of disease	Individuals who died as a result of the risky behavior	1.66 (1.04–2.66)
Bak et al. (93)	Yes/Yes/Yes	South Korea	Case-control	CLIA	IgG	P: (18, 80) C: (22, 59)	P: (F:69, M:66) C: (F:80, M:75)	Yes	Healthy	Depressive symptoms	2.49 (1.06–5.82) Seropositivity: 21/155 (14%) v. 8/135 (6%) High antibody titers (>150 IU/ml): 7/21 (33%) v. 2/8 (25%)
Fond et al. (99)	No/Yes/Yes	France	Cohort	EIA (S)	IgG	(32.0 ± 8.6)	(F:66, M:184)	No	Schizophrenia	Schizophrenia	1.27 (0.26–6.25)
Burgdorf et al. (94)	Yes/Yes/No	Denmark	Case-control	ELISA (S)	IgG	P: (37.4) C: NA	P: (F:377, M:278) C: (F:NA, M:NA)	No	Blood donors (psychiatric disorders without registered suicide attempt)	Blood donors (psychiatric disorders with registered suicide attempt)	1.25 (1.04–1.49) Seropositivity: 193/655 (29%) v. 1,633/6,503 (25%) Prospective subgroup; outcome after blood sampling: 3/23 (13%) v. 1,319/5,259 (25%)
Sari and Kara (102)	No/Yes/No	Turkey	Case-control	ELISA	IgG IgM	P: (12, 17) C: (12, 18)	P: (F:43, M:7) C: (F:43, M:7)	No	Healthy	Psychiatric disorders	7.44 (0.37–147.92)
Yalin Sapmaz et al. (103)	No/Yes/No	Turkey	Cross-sectional	ELISA	IgG IgM	(15.6 ± 1.59)	(F:31, M:6)	No	Depression	Depression	94.50 (7.45–1198.62)

*C, control; CI, confidence interval; CLIA, chemiluminescent immunoassay; EIA, enzyme immunoassay (S): solid-phase EIA; ELISA, enzyme-linked immunosorbent assay (S): solid-phase ELISA; F, female; IF, indirect immunofluorescence; IgG, immunoglobulin G; IgM, immunoglobulin M; IU, International unit; M, male; mL, milliliter; OR, odds ratio; P, patient; PANSS, Positive and Negative Syndrome Scale; S.D., standard deviation; SDV, self-directed violence; SES, socioeconomic status; v., versus*.

a *Data of cases are presented first, control population second*.

### Sutterland et al. 2019

The systematic review guidelines from the Preferred Reporting Items for Systematic Reviews and Meta-Analyses (PRISMA) statement (79) were applied in this meta-analysis (80) that combined 14 studies on suicide attempt/suicide. A systematic search was done throughout EMBASE, Medline, and PsychInfo till February 11, 2019 (Prospero #CRD42018090206). Inclusion criteria were: (i) original scientific studies in any language; (ii) having comparable quantitative data; (iii) analyses of latent *T. gondii* infection via any of the following assays that measure IgG antibodies: enzyme-linked immunosorbent assay (ELISA) or immune fluorescence, Sabin–Feldman dye test, immune hemagglutination, or complement fixation; (iv) cohort or case–control studies with human subjects; and (v) data on suicide attempts. Exclusion criteria were: (i) no control group; (ii) case series or case reports; and (iii) inclusion of immunocompromised individuals. Three researchers performed screening of search results by examining abstracts and manuscript titles. The Cochrane criteria of quality for cohort or case–control studies (81) were followed to screen study quality, independently by two researchers. The type of controls (healthy controls vs. psychiatric controls) without suicide attempt history were criteria for stratification of analyses. Studies were classified as having a prospective design when measurement of *Toxoplasma* antibodies preceded the behavioral outcome in a longitudinal cohort, or when, in a cross-sectional study, the antibody measurement followed shortly after the suicide attempt. Another grouping was based on studies that included only schizophrenia patients vs. studies that included other diagnostic categories.

For seropositivity definition, the reported criteria in each study were used, and if multiple methods were reported, the ones that used the smallest effect size were analyzed. Serointensity (antibody titers) were also analyzed as either reported by individual studies or deduced from average antibody titers reported in the study.

An odds ratio (OR) was computed for all studies, with utilization of ORs adjusted for confounders, when accessible. All analyses used random effect modeling. Eyeballing and calculating *I*^2^ were used to estimate heterogeneity among studies. Comprehensive Meta-analysis Software 3.0 (82) was used for meta-analytical calculations. For better estimation of the true ORs, when applicable, Duval and Tweedie's trim and fill method was used. Egger's test (significant if the one-sided *p* < 0.10) and examination of funnel plots were used to estimate the potential for bias. The latent *T. gondii* global prevalence (Pglob) was estimated to be 30% (83), and the population attributable fraction (PAF) was calculated using the formula: PAF = [Pglob × (RR – 1)]/[Pglob × (RR – 1) + 1] (84), where RR is the Risk Ratio (at low frequencies identical to Odds Ratio) (85). To estimate the degree by which the moderators altered variance of the main effect, the moderators were analyzed as covariates using regression analysis with methods of moments for continuous variables and mixed-effects analysis for categorical variables. For each specific moderator *R*^2^ was calculated.

#### Results

**Figure 2:**
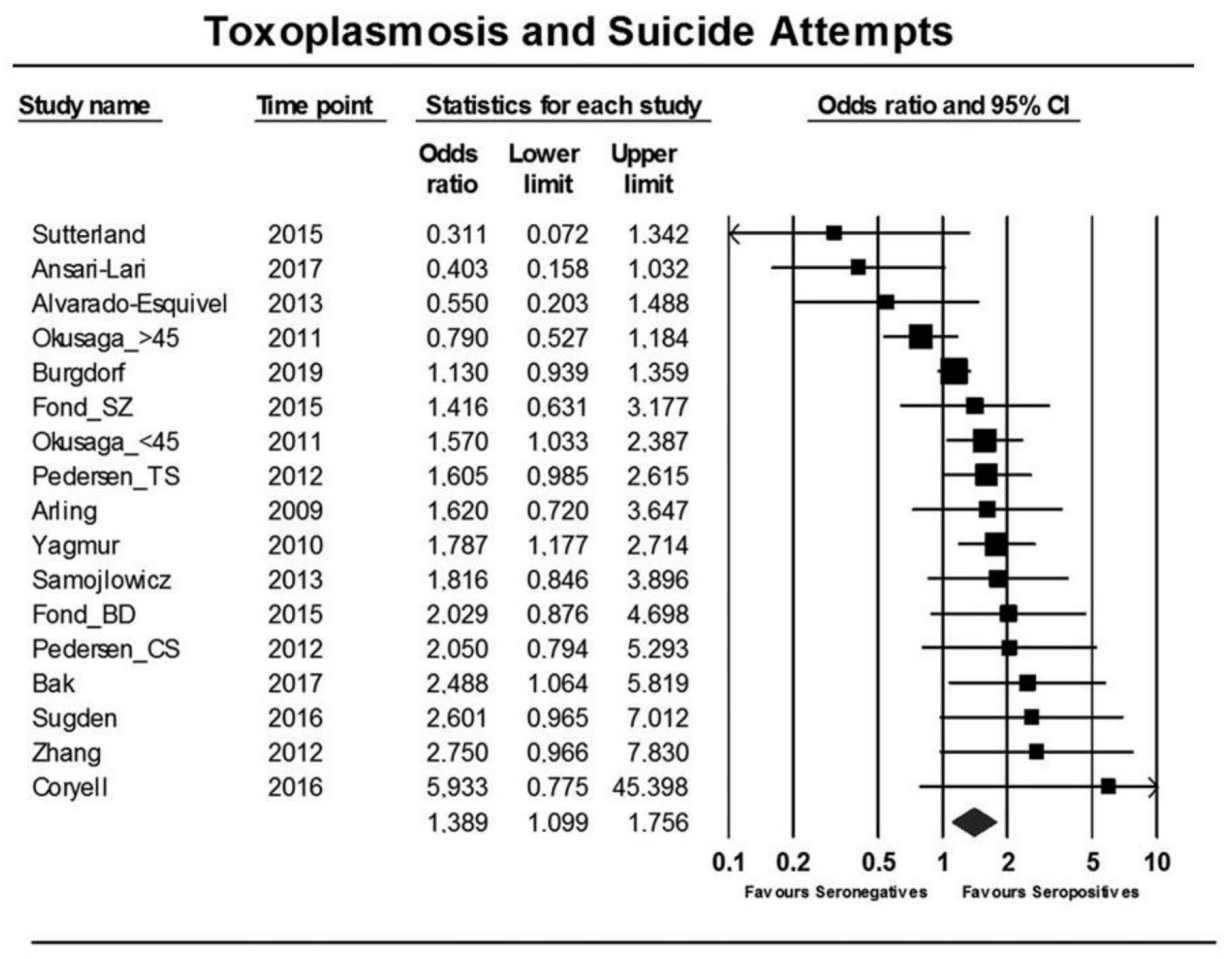
Forest plot showing an association between *T. gondii* infection and suicide attempts as reported in a meta-analysis by Sutterland et al. (80) using the random effects model. Serointensity has not been presented, and, thus, the first study on *T. gondii* infection and suicide attempt appears as negative [Modified and reprinted with permission from (80); Copyright (2019); with permission from Cambridge University Press; License # 4963421068137].

An overall significant OR (for suicide attempt) of 1.39 [95% confidence interval (CI): 1.10–1.76, *p* = 0006] (see Figure 2) was associated with *T. gondii* infection (seropositivity) in the thirteen published studies, that evaluated the association between suicide attempt/death by suicide and latent *T. gondii* infection and one study that was not published (74–78, 86, 87, 89–94). The Egger's test did not identify bias (*p* = 0.15). Additionally, similar and significant OR was rendered by Duval and Tweedie's trim and fill analysis. Considerable heterogeneity among studies was identified (*I*^2^ = 55%, *p* = 0.003, τ^2^ = 0.103). Two main sources of heterogeneity have been identified—the diagnostic composition of the samples (schizophrenia only vs. other diagnoses) and the nature of controls (psychiatric vs. normal controls). In samples consisting of individuals with a diagnosis of only schizophrenia, no significant elevation in OR for suicide attempt was observed in *T. gondii*-positive cases, but in samples of individuals with mixed psychiatric disorders, the association was significant [OR = 1.8 (95% CI: 1.44–2.24), *p* < 0.001]. In turn, when psychiatric (non-attempter) controls were used, the association was not significant, while when healthy controls were used, the association was robust and significant [OR = 1.9 (95% CI: 1.48–2.44), *p* < 0.001].

In regard to serointensity, only eight studies were identified, starting with Postolache group's first study—(74, 76–78, 86, 89, 92, 93), rendering an overall OR of 1.22 (95% CI: 0.96–1.55, *p* = 0.11), with no significant results in schizophrenia [OR = 0.99 (95% CI: 0.77–1.29)] but significant in mixed diagnostic samples [OR = 1.66 (95% CI: 1.29–2.12, *p* < 0.001]. A high heterogeneity among studies was identified (*I*^2^ = 62%, *p* = 0.004, τ = 0.252). When investigating potential sources of heterogeneity, a robust and significant diagnostic effect emerged (*R*^2^ = 61%, *p* = 0.004) with no significant associations in studies on participants with schizophrenia [OR = 0.99 (95% CI: 0.77–1.29)] vs. various other psychiatric disorders [OR = 1.66 (95% CI: 1.29–2.12), *p* < 0.001].

Population attributable fraction: If the average infection rate of *T. gondii* in humans globally is assumed to be 30%, the computed PAF [0.3 × (OR – 1)]/[0.3 × (OR – 1) + 1], showed that in theory if *T. gondii* infection can be totally prevented, suicidal behavior would decrease by ~10% (95% CI: 3–19%).

#### Relevance

This is the first meta-analysis confirming an association between suicide attempts and *T. gondii* serology, as we had originally reported. The association of *T. gondii* serointensity with suicide attempts in samples that did not include schizophrenia patients exclusively, confirmed the very first link between *T. gondii* serology and suicide attempts that the Postolache group and their collaborators had identified in patients with recurrent mood disorders (74). Regarding the uncovered effect of psychiatric diagnosis, with insignificant associations between *T. gondii* serology and suicide attempts in schizophrenia in the Postolache group's individual study, only the schizophrenia patients that were younger (23) or those with the plasma tryptophan's metabolite kynurenine in the highest quartile (95) manifested an association between *T. gondii* and suicide attempts. It is possible that the link between *T. gondii* and schizophrenia is stronger (54) than its association with suicidal behavior and that the initial elevation in suicide risk in response to the diagnosis and early losses associated with the illness is a much more impactful clinical phenomenon. It may also be possible that other aspects outweigh the potential suicide risk elevation by *T. gondii* infection in schizophrenia patients. For instance, in schizophrenia patients, *T. gondii* serology interactive biomarkers (such as monoamine metabolites) are also associated with other independent suicide risk factors, including autoimmune markers [such as gliadin antibodies (96)], or smoking (97), that may override in magnitude the strength of *T. gondii* associations. In regard to psychiatric vs. healthy controls, the lack of significance when psychiatric controls were used suggests several possibilities—it is possible that despite impressions at an individual study level (where the associations have been robust to adjustment for psychiatric illness), some degree of mediation via mental illness, or perhaps confounding by severity of mental illness cannot be ruled out. Nevertheless, in our collaborative study (78), the associations between *T. gondii* and subsequent suicide attempts were robust with adjustment for baseline mental illness (and even parental history of mental illness). Additional *T. gondii*-related variables, such as strain and the method of infection (at present it is possible to test for an oocyst targeting IgG antibody) require further research.

The calculated PAF is epidemiologically and clinically meaningful, with 1 in 10 suicide attempts being averted if *T. gondii* infection is completely prevented or chemically eradicated. Identifying the characteristics of specific *T. gondii* positive individuals with recurrent suicide attempts who may benefit the most from interventions geared to prevent reactivation is an important aim of a future preliminary study.

### Soleymani et al. 2020

This systematic review and meta-analysis (98) followed the PRISMA guidelines (79) and presents combined results of 15 studies. The authors searched Institute for Scientific Information (ISI), Medline, and Scopus, and the reference list of selected studies for case-control, cohort, and cross-sectional studies that investigated associations between suicidal behavior (as outcome) and *T. gondii* infection (as predictor). Egger and Begg tests were used to evaluate publication bias. *I*^2^ statistics and chi-square tests were used to assess heterogeneity among studies. For combining results, a random effect approach was used.

#### Results

**Figure 3:**
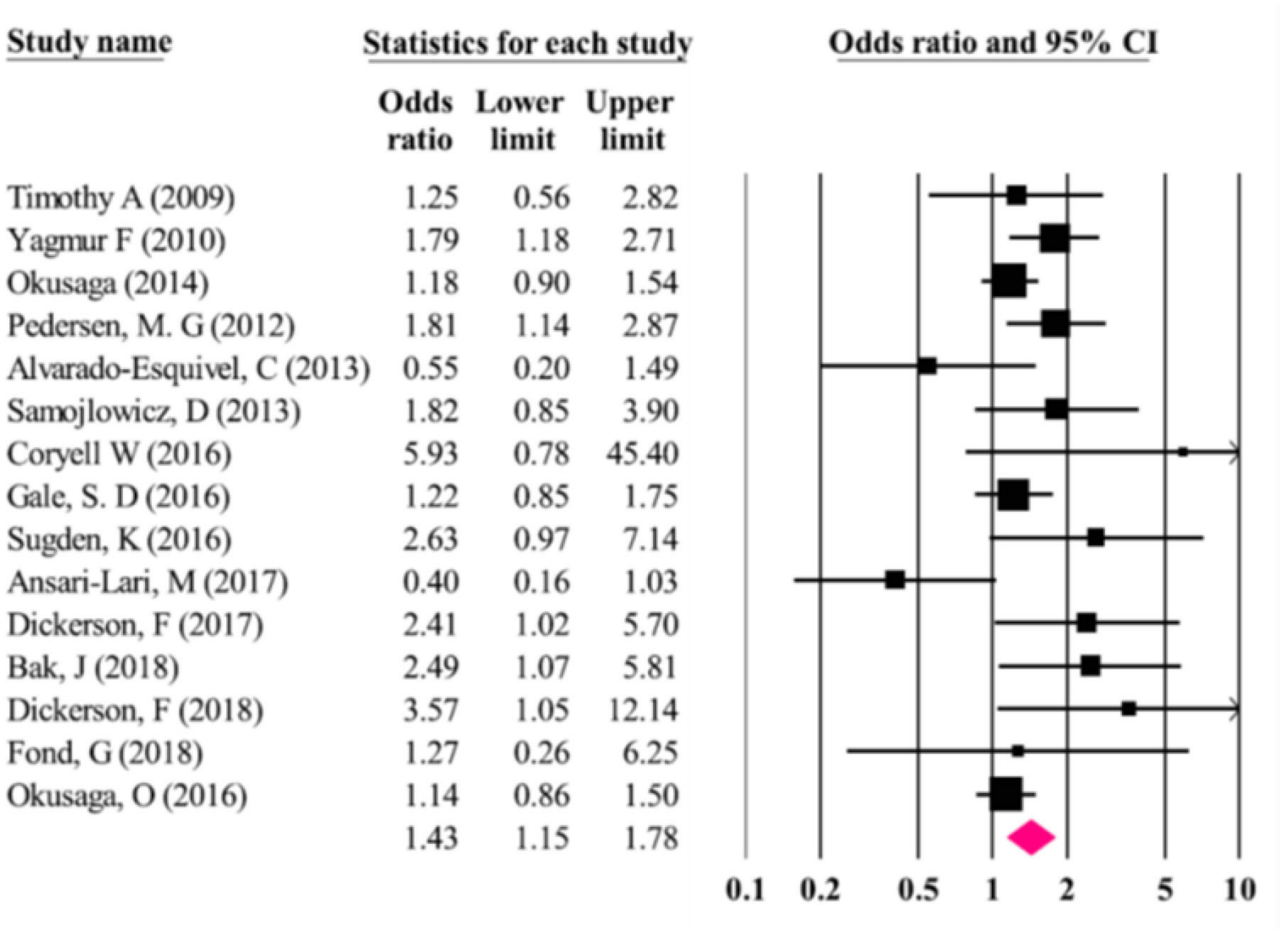
Results (odds ratios and 95% confidence intervals) from the individual studies and the meta-analysis by Soleymani et al. (98). The first study from the Postolache group, by Timothy Arling is wrongly identified as Timothy et al. instead of Arling et al. (74). Furthermore, studies that have identified associations between serointensity (but not seropositivity), such as Arling et al. (74) and Okusaga et al. (95) appear as carrying no significant association [Modified and reprinted with permission from (98); Copyright (2020); with permission from Springer Nature; License link: http://creativecommons.org/licenses/by/4.0/].

Odds of suicidal behavior were higher in *T. gondii* seropositive vs. seronegative individuals [OR = 1.43 (95% CI: 1.15–1.78)] (see Figure 3). No publication bias was identified (Egger and Begg test: *p* = 0.28). The *I*^2^ test demonstrated a moderate heterogeneity (*I*^2^ = 0.71) leading to the choice of random-effect modeling to perform the meta-analysis.

#### Relevance

The Soleymani et al. (98) meta-analysis analyzed several studies [for example (99–101)] not included in Sutterland et al. (80) and Amouei et al. (105) meta-analyses, but also left out several studies that were included in those meta-analyses [e.g., (77, 94, 102–104)]. It errs in naming suicide attempts as suicides, and it phrases the conclusion in a causal way. Additionally, parasuicides were also included in the “suicide” category, leading to a broader inclusion of self-directed violence—i.e., suicidal and non-suicidal, lethal and non-lethal. Nevertheless, despite its unprecise definition of outcome and only partially overlapping study selection, the study still confirmed the significant association with *T. gondii*, and yielded an effect size similar to the previous meta-analysis of Sutterland et al. (80).

### Amouei et al. 2020

This meta-analysis (105) evaluated the potential association of *T. gondii* with the risk of suicidal ideation and suicide attempts. PRISMA guidelines (79) were followed and the protocol was enlisted with The International Prospective Register of Systematic Reviews (PROSPERO). “Google Scholar,” “PubMed,” “ScienceDirect,” “Scopus,” “Web of Science,” “PROSPERORegister,” “EMBASE,” “CINAHL,” and “ProQuest” were searched, without language restriction. Five studies on suicidal ideation and 22 studies on suicide attempts were combined. In addition to the added focus on suicidal ideation, this meta-analysis also analyzed *T. gondii* immunoglobulin M (IgM), and not only IgG.

The quality of selected studies was checked with the standard checklist of the Strengthening the Reporting of Observational Studies in Epidemiology (STROBE) (106). Statistical heterogeneity among studies was evaluated using Cochran's Q test (represented as chi-square and *p*-values) and index *I*^2^ representing percentage of variability due to heterogeneity (107). The study planned to use random effects modeling if the heterogeneity was significant or high (*p* < 0.05 or *I*^2^ > 50%). The analysis used random effects models. A meta-regression analysis was used for separately evaluating the contribution to heterogeneity of moderators represented by differences in sub-groups based on the type of study, target population, control population, detection method, and continent. For univariate meta-regression and multivariable meta-analysis, *R*^2^ values were calculated to express the effects of moderators as covariates (specifically the amount of heterogeneity in the meta-regression and meta-analysis that can be explained by the moderator variables). Funnel plots were used to illustrate the risks of bias and the Egger's regression test was used to evaluate the presence of bias (positive for *p* < 0.10) (108). For a better estimation of true OR, when applicable, the Duval & Tweedie non-parametric “fill and trim” linear random method was used. To estimate the possibility that one single study is responsible for the meta-analytic results, a sensitivity analysis using the “omitting one” method was implemented.

#### Results

**Figure 4:**
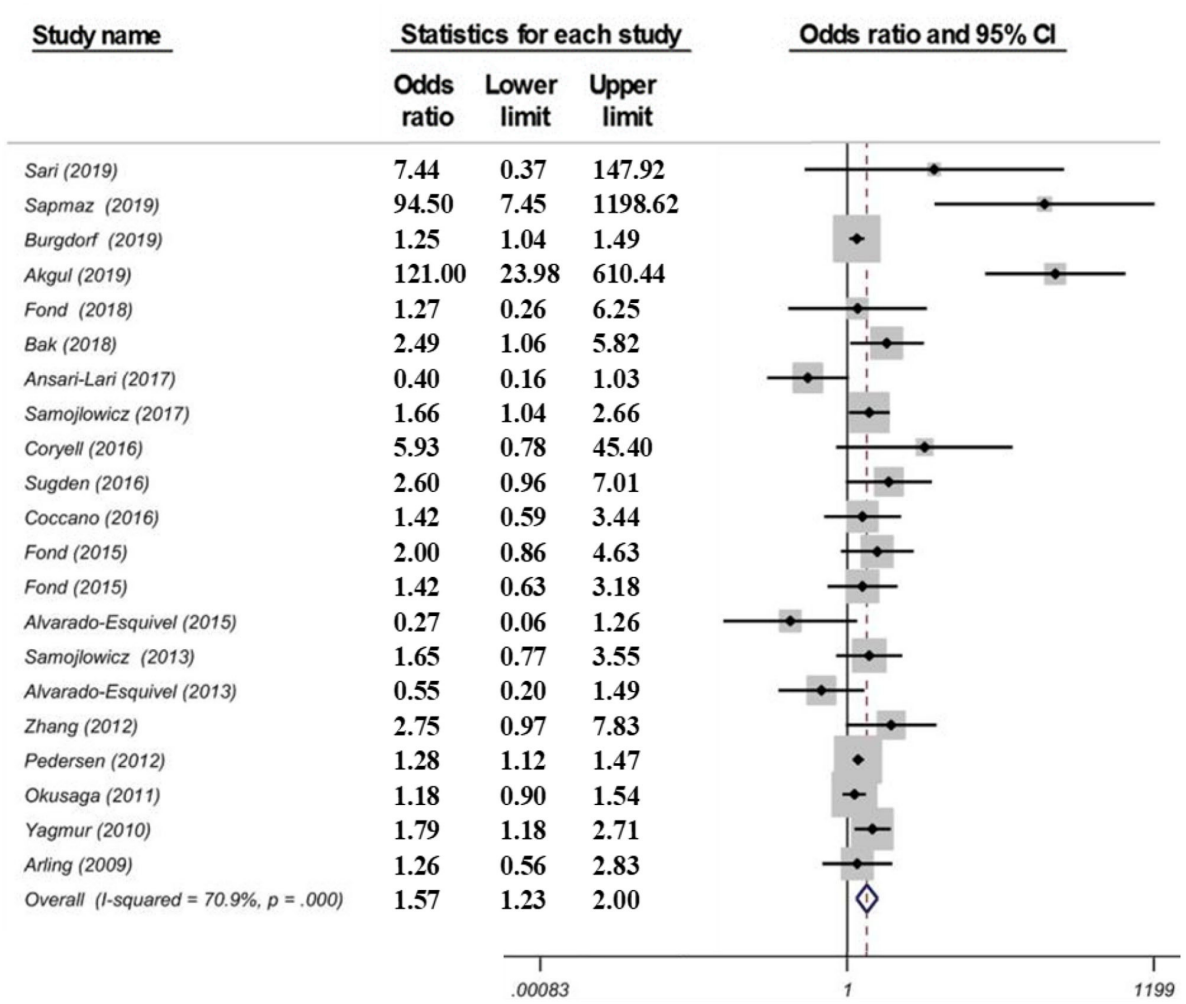
Forest plot showing a correlation between *T. gondii* infection (IgG) and suicide attempts as reported in a meta-analysis from 21 data sets by Amouei et al. (105). Serointensity associations with suicide attempts, even when significant, such as Arling et al., Okusaga et al., and Zhang et al. are not presented and studies thus appear as carrying no significant association with *T. gondii* serolog*y* [Modified and reprinted with permission from (105); Copyright (2020); with permission from John Wiley & Sons; License # 4963420399853].

Suicidal ideation was not significantly associated with *T. gondii* IgG seropositivity [OR = 0.90 (95% CI: 0.42–1.94)] based on a random effects application. The Egger's test suggested no significant bias (*p* = 0.86), the Duval and Tweedie's trim and fill model provided the same ORs, and the “leave one out” sensitivity analysis showed that no single study substantially influenced the negative result. For suicide attempts, there was a significant association with *T. gondii* IgG seropositivity [OR = 1.57 (95% CI: 1.23–2.00)] (see Figure 4), but not with IgM seropositivity [OR = 1.41 (95% CI: 0.78–2.54)]. There was no evidence of publication bias (Egger test; *p* = 0.25). The “leave-one-out” sensitivity analyses confirmed the robustness of the findings. A high level of heterogeneity was noted (χ^2^ = 68.72, *p* = 0.000, *I*^2^ = 70.9%). A univariable meta-regression to identify the source of heterogeneity failed to identify a univariate significant moderation. Specifically, the location/continent, the study design (β = 0.31, *p* = 0.31, *R*^2^ = 9.90), the control population (β = −0.52, *p* = 0.07, *R*^2^ = 8.19), the method of diagnosis (β = 0.34, *p* = 0.18, *R*^2^ = 17.35), or the target population (β = −0.22, *p* = 0.39, *R*^2^ = 20.30) exerted no significant effect on the heterogeneity among studies. However, when the combined effect of these variables was analyzed, interactively, the continents (β = 0.66, *p* = 0.04), the study design (β = 0.84, *p* = 0.01) and type of control group (β = −2.84, *p* = 0.03) had a significant effect on suicide risk. When all moderators were entered simultaneously in the multiple regression model as covariates of the true effect, they explained 34.7% of the variance (τ^2^ = 0.48, *R*^2^ = 0.347) of the main effect.

#### Relevance

The relevance of this third meta-analysis is that it is larger than previous meta-analyses and it also included suicide ideation studies rather than only suicide attempts, and *T. gondii* IgM rather than IgG positivity studies only. There was no significant association between suicide ideation and *T. gondii* IgG (five studies) and IgM (three studies) positivity. Even if in the primary papers, a strong association between IgM positivity and suicidal behavior was reported (OR = 2.41) (100), no significant meta-analytic association between IgM positivity and suicide attempt emerged. The relevance of IgM relies on the current understanding, a departure from the past, that it may reflect not only an acute infection, but also infection with a new serotype or reactivation of the parasite (109). The association between *T. gondii* IgG seropositivity and suicide attempt was stronger than in previous meta-analyses [OR = 1.57 (95% CI: 1.23–2.00)].

### Summary of Meta-Analyses

The three recent meta-analyses converge in supporting a moderate association between *T. gondii* IgG seropositivity and suicide attempt. In sum, the odds of suicide attempt are between 39 and 57% higher in *T. gondii*-IgG positive individuals. The three studies used similar methods with all using the PRISMA guidelines. All studies used random effects modeling. Quality was determined by different methods—Sutterland et al. (80) used the Cochrane criteria of quality on case–control or cohort studies (81), Amouei et al. (105) used Strengthening the Reporting of Observational Studies in Epidemiology checklist (STROBE) to assess the quality of selected studies (106), and Soleymani et al. (98) used the Newcastle and Ottawa statement (NOS) checklist (110). Sutterland et al. (80) and Amouei et al. (105) share the use of Egger test and visual inspection, while Soleymani et al. (98) reported the Begg test (111). *I*^2^ was used by all studies to estimate heterogeneity among studies. There were also unique features: Sutterland et al. (80) analyzed serointensity and Amouei et al. (105) analyzed suicidal ideation and not only attempt, *T. gondii* IgM antibodies and not only IgG antibodies, and was the only one to perform an omitting one sensitivity analysis.

Limitations include the lack of information on the socioeconomic status among groups, potentially as a confounding variable contributing to both infection and suicidal behavior, and limited information on IgG avidity, immune activation and *T. gondii* serotypes. The diagnosis of patients (schizophrenia vs. others), the nature of control participants (healthy vs. psychiatric), the location of the study, and study design (e.g., cross-sectional vs. longitudinal) may have contributed to heterogeneity of findings among the different meta-analyses. Major differences exist between continents and countries in regard to the prevalence of Toxoplasmosis (112), likely contributing to heterogeneity (113). Additionally, many medical conditions (“disease burden”) had disability-adjusted life years (DALY) or mortality correlate with *T. gondii* seroprevalence in an ecological approach with minimum adjustment (for GDP only), and thus, tentatively representing potential “hidden variables” for the *T. gondii*–suicide association. However, many conditions associated with *T. gondii* infection manifested opposite correlations in European vs. non-European countries (113). In addition to the specific dominant serotype, dominant way of infection (oocyst vs. tissue cyst), exposure to gut, respiratory, indoor and outdoor flora, especially during critical time intervals during early childhood, may lead to long-term contributory, neutral or even protective outcomes from *T. gondii* infection. For instance, asthma was negatively correlated with *T. gondii* prevalence in European countries (low statistical trend) and positively correlated in non-European countries. Similarly, suicide had positive low-grade associations in European countries and negative associations in non-European ones. This could not be explained as differential association of *T. gondii* with violence in general, as violence was positively associated with suicidal behavior across the continents (113).

As an informed working hypothesis for future studies, non-psychotic patients compared to a healthy control group, using a prospective study design (antibodies measured first, or if second, after a very brief interval after attempt), and a higher lethality attempt will more likely yield a stronger effect size of *T. gondii* associations with suicide attempts. This information may be of significant interest for future randomized clinical trials.

## Searching for Potential Mechanisms of the Uncovered Association, Even Before Establishing Causality

After identifying significant links between *T. gondii* seropositivity and suicidal behavior in patients with mood disorder, schizophrenia, acute attempters, and mothers (post-delivery), we then went on to analyze intermediate phenotypes of suicidal behavior rather than suicide attempts. We reported, for the first time, age- and gender-specific associations between trait impulsivity and aggression and *T. gondii* status in 1,000 super-healthy individuals with no personal and parent family history of mental illness or suicidal behavior (limiting the collision between personality traits, genetic and early developmental influences on mental illness, and psychiatric treatment) (114). Further, we analyzed interactions between *T. gondii* serology and the plasma monoamine precursor for serotonin and kynurenine (i.e., tryptophan) in predicting suicidal behavior in schizophrenia patients. We found that coexistence of a high plasma kynurenine (top quartile) and positive *T. gondii* IgG serology were necessary for a significant link between *T. gondii* and history of suicidal behavior in persons with a diagnosis of schizophrenia (95). In the endophenotypic direction (impulsivity, aggression), elevated levels of plasma phenylalanine/tyrosine (Phe:Tyr) ratios (specifically in the top quartile)—precursors of catecholamines including dopamine—interacted significantly with *T. gondii* seropositivity in younger males only in predicting impulsivity (115). Additionally, *T. gondii* seropositivity moderated the association between Phe:Tyr ratio and aggression (i.e., only in the *T. gondii* seropositive group—an association between a higher ratio between Phe:Tyr and aggression was significant) (116). The associations between *T. gondii* seropositivity and both impulsivity and aggression (self-report and observed) were further confirmed in a psychiatric population with high levels of explosive impulsive aggression, i.e., individuals with Intermittent Explosive Disorder (117).

### Clinical Syndromes Potentially Connecting *T. gondii* With Suicidal Behavior

Various cognitive and neuropsychological abnormalities, as well as executive function deficits, are associated with suicidal behavior (118–125). A number of studies have identified links between *T. gondii* and progressive cognitive deficits in humans (126–131), although there are also negative reports (132). In addition to the general low-grade immune activation by *T. gondii* and priming of immune cellular substrates in the brain that may represent the most common underlying mechanism, there is also a more novel hypothesis based on pathogen-mediated N-methyl-D-aspartate (NMDA) receptor autoimmunity and barrier dysfunction (133).

### Decision-Making Deficits and Suicidal Behavior

Data suggest that, for some, suicidal behavior is linked to deficits in cognitive functioning that negatively impacts planning, regulation of goal directed behavior, and strategic decision-making (134, 135). For example, work by Jollant et al. showed that those with a history of violent suicide attempts demonstrated deficits on the Iowa Gambling test, a test of decision-making in an emotionally charged context (136, 137). Deficits of decision-making represent both a state and especially trait (and endophenotype) markers in suicidal behavior (138–144). These findings are supported by results suggesting that those with a history of suicide attempts perform in a manner that prioritizes immediate rewards while discounting future consequences (“delayed discounting”) (145). Finally, work by Jung et al. which demonstrates widespread yet discrete changes in both functional brain networks and interconnectivity, corroborates previous findings regarding cognitive dysfunction among those at increased risk for death by suicide (146).

The rodent versions of the Iowa Gambling Task exhibit good construct and face validity (147, 148). This task in rats is modulated by the serotonin transporter level, as shown before in humans (149). Furthermore, in a rat chronic pain model (150), Iowa Gambling Task impairment was associated with a reduction in 5-hydroxyindoleacetic acid in the orbitofrontal cortex.

In rodents, latent *T. gondii* infection reverses innate fear of cat odor, and other stimuli that precede predation (49). The reduced fear and anxiety-like behavior reported in infected rodents may be the result of dendritic retraction in the basolateral amygdala, lower corticosterone secretion (151), and epigenetic modulation in the medial amygdala (152). Evidence suggests alterations in decision-making in rats infected with *T. gondii*, specifically induction of effort aversion by the parasite (153).

Although there is literature on latent Toxoplasmosis and impulsivity in healthy humans (114) and individuals with Intermittent Explosive Disorder (117), there is no report, to our knowledge, on decision-making deficits. We have just completed a 5 year study on Veterans, with Iowa Gambling Task measurements in suicide attempters and controls, with and without IgG markers of *T. gondii* infection. Results are pending. If they are able to confirm that impaired decision-making, a known risk for suicidal behavior, is linked to chronic infection with *T. gondii*, and possibly that decision-making impairments mediate or moderate the *T. gondii*-induced elevation of risk of suicidal behavior, then impaired decision-making could potentially become an endophenotypic marker of suicide-risk associated with *T. gondii* infection.

### Sleep and Wake Abnormalities

Considering that sleep abnormalities are more readily correctable risk factors for suicidal behavior (15), and that sleep is dysregulated by dopamine and NMDA receptor stimulation (increased in chronic *T. gondii* infection), as well as by low-grade immune activation (present in Toxoplasmosis), and because sleep deprivation alters decision-making and elevates suicide risk, we hypothesized that sleep abnormalities mediate the association between latent infection with *T. gondii* and suicidal behavior. If so, we expected that sleep abnormalities would be abundantly identified in *T. gondii* IgG positives in a relatively large number (*N* = 833) of participants with high seroprevalence of *T. gondii*, such as Old Order Amish. This would have then provided a potential targetable behavioral aim to reduce suicide risk in *T. gondii*-infected individuals, via treating their sleep abnormalities. However, our results were disappointing, with no significant detrimental association of sleep problems with *T. gondii* serology. In fact, *T. gondii*-seropositive Amish individuals reported less sleep problems and daytime problems due to poor sleep, and longer, rather than shorter sleep duration, with earlier mid-sleep time and bedtime (154). It is possible, just as with allergies, that certain strains or mode of infection (e.g., tissue cyst vs. oocyst) of *T. gondii* in specific undetermined conditions and for certain phenotypes, serves as a microbial “Old Friend” —modulating immune responses, and thus, reducing downstream effects of chronic low-grade immune activation.

In a study on Old Order Amish, the Postolache group reported an association between trait hopelessness and *T. gondii* IgG serointensity (155) (which could reflect a more widespread infection, or a more reactivating or neurotropic course (see section Hopelessness and *T. gondii* Serointensity in Old Order Amish).

In conclusion, while the decision-making deficits, other cognitive abnormalities, and trait hopelessness could mediate *T. gondii* predictive links with suicidal behavior, this requires confirmation in targeted experiments. Additionally, it is highly unlikely that sleep disturbances mediate the predictive association between *T. gondii* and suicidal behavior.

### Potential Molecular Mechanisms Linking *T. gondii* With Suicidal Behavior

Immune dysregulation is a major candidate mediator of the association between *T. gondii* and suicidal behavior. A low-grade immune activation is necessary to contain the parasite to its slow-growing form inside tissue cysts, with pro-inflammatory cytokines playing a central role (156, 157), through activation of microglia and monocyte-derived macrophages trafficking to the central nervous system (CNS) (158) and activation of lymphocytes and macrophages in the periphery (159). Moreover, the intermittent immune escape and reactivation of *T. gondii* results in an immune response to tachyzoite formation, and local and systemic invasion leading to more pronounced elevations in molecular mediators of immune activation. For instance, higher peripheral levels of tumor necrosis factor (TNF) and interleukin 6 (IL-6) in suicide attempters relative to healthy controls and non-suicidal depressed patients have been previously reported (160). Similarly, there have been reports of *T. gondii*-seropositive individuals having elevated levels of IL-6 (161); TNF has an active role in controlling *T gondii* replication (162), and was reported to be elevated in *T. gondii*-positive women (66) and in suicide attempters (160).

Findings relating immune activation with *T. gondii* and suicidal behavior would need to account for mental illness, either through design (comparison groups, inclusion/ exclusion) or through adjustments, as more literature emerges regarding inflammation and mental illness, including major depressive disorder (163–165), bipolar disorder (163, 166, 167), and schizophrenia (163, 168–171), as well as treatment of mental illness (172–175). In both animal studies (176–179) as well as human literature (180–184), behavioral dysregulation commonly observed among those with mental illness, such as trait impulsivity (185, 186) and aggression (187–190) that are described as intermediate phenotypes for suicidal behavior (12, 191, 192), have been also positively associated with higher levels of inflammation.

To reduce exposure to mediators of the immune system, the parasite hides within cystic structures inside glial cells and neurons. Cysts containing *T. gondii* primarily concentrate in brain regions involved in fear induction, such as the amygdala, and fear modulation, such as the prefrontal cortex (49, 193, 194). Particularly, chronic infection with *T. gondii* potentially contributes to the reduced fear and anxiety-like behavior by inducing dendritic retraction in the basolateral amygdala (151). Two genes coding for tyrosine hydroxylase (rate-limiting enzyme involved in dopamine synthesis) (195) are present in the *T. gondii* genome. It has been reported that when stimulated, PC12 cells infected with *T. gondii* release greater levels of dopamine (48). Neurotoxicity and increased arousal are potential consequences of dopamine increase, further contributing to increased risk of suicidal self-directed violence (SSDV). Throughout the lifetime of immunocompetent individuals, *T. gondii* continues to live in a slow-growing form called bradyzoite. Yet, intermittent reactivations occur in states of relative immunosuppression, which has been specifically proposed to be one mechanism by which psychiatric episodic manifestations and exacerbations, as well as episodes of self-directed violence, could occur during immunosuppression (196, 197).

An important mechanism of resistance for the host is the relative tryptophan deprivation of the microorganism through degradation of tryptophan toward kynurenines, mediated by activation of the enzyme indoleamine 2,3-dioxygenase (IDO) by pro-inflammatory cytokines (157). Activation of IDO also elevates production of kynurenine pathway metabolites, quinolinic acid (QUIN) and kynurenic acid (198), known to be potent neuromodulators (199) of NMDA receptors. Mice with chronic *T. gondii* infection have a 7-fold increase in brain kynurenic acid content and a smaller increase in kynurenine levels, implying an activation of kynurenine pathway enzymes (200).

Kynurenine findings may be highly relevant for the associations between infection and suicidal behavior and between inflammation and suicidal behavior. The collaborative work of our group at the University of Maryland with Dr. Mann's group at Columbia University led to the first report of elevated plasma kynurenine levels in individuals with a diagnosis of major depression, with vs. without history of suicide attempts (29). Particularly, cerebrospinal fluid (CSF) kynurenic acid concentrations were found to be associated with increased IL-6 levels and violent suicide attempts (201). In Postolache team's collaborative study with Lena Brundin's group at the University of Lund, CSF QUIN levels were found to be elevated in individuals with recent non-fatal suicidal self-directed violence (NF-SSDV) (in particular, those with more severe SSDV), independent of psychiatric diagnosis, with normalization of these values 6 months after a suicide attempt that had led to a hospitalization (202). Consistently, increased postmortem counts of QUIN-reactive microglial cells in suicide victims was found in the anterior midcingulate cortex (MCC) and subgenual anterior cingulate cortex (sgACC) of suicides (203). QUIN is an excitotoxic NMDA receptor agonist, and potentially, its elevation in individuals with *T. gondii* infection may represent, in the future, a more targeted application of ketamine that shows a robust anti-suicidal effect within minutes or hours after intravenous administration (204, 205).

Elevation of titers of *T. gondii* IgG may reflect a more recent infection, more virulent infection, or a more extensive infestation. A more recent reactivation or more frequencies of activation may also be possible explanations. Furthermore, molecular mimicry may lead to a direct effect of the IgG antibodies. IgG antibodies against *T. gondii*, as well as other infectious agents, may cross-react with epitopes in neural tissue (206, 207). This remains speculative, as it has not been studied specifically for *T. gondii*.

Effects of *T. gondii* infection on the homeostatic interactions between the gut microbiota and gastrointestinal mucosa may provide an alternative pathway for *T. gondii* elevating the risk of suicide. Acute *T. gondii* infection induces gastrointestinal inflammation that is dependent on CD4+ T lymphocytes located in the lamina propria, mediated by pro-inflammatory cytokines and by subepithelial bacterial translocation (208) and increased gut permeability. Infection with *T. gondii*, despite its transient passage through the gut during acute infection, has long-term effects on mucosal immunity, resulting in activation of microbiota-specific T cells and loss of tolerance to gut commensal bacteria (209). It has been suggested that intestinal inflammation induced by *T. gondii* bears a resemblance to inflammatory bowel disease (IBD), especially Crohn's disease (210), and conversely, anti-*T. gondii* antibodies are increased in individuals with IBD (211). Moreover, IBD is a well-known risk factor for suicide (212).

We will now revisit in more detail several of the Postolache team's studies on *T. gondii*, specifically: (1) the first study in mood disorder; (2) the first prospective study; (3) the study in schizophrenia in which we intersected *T. gondii* with plasma kynurenine levels; (4) the study in healthy individuals relating intermediate phenotypes for suicidal behavior to *T. gondii;* and (5) the intersection of Phe:Tyr ratio and *T. gondii* seropositivity in relationship to impulsivity.

## The First Report: *T. gondii* Serology in Individuals With Mood Disorders

The Postolache team and their collaborators from Johns Hopkins University and Sheppard Pratt were the first to test the specific hypothesis of an association between *T. gondii* IgG (serointensity and seropositivity) and suicidal behavior (74). We specifically hypothesized that *T. gondii* serology is positively associated with having attempted suicide in the past and with number of attempts, the strongest predictor of suicide. We tested our hypotheses using samples obtained from ongoing studies on potential environmental triggers of depression exacerbation and suicide attempts in individuals with recurrent mood disorders.

*T. gondii* serology was compared between suicide attempters (all with history of mood disorders: 99 participants) with two control groups—psychiatric control (history of mood disorders without history of suicide attempt; 119 participants) and a healthy control group (39 individuals). A Structured Clinical Interview for DSM IV was the basis for establishing diagnosis. This was an analysis of existing clinical and behavioral data, and analysis of blood samples collected from two other studies—one from a study on potential environmental triggers of depression exacerbation and allergens, and one from a study on suicide attempts in individuals with recurrent mood disorders.

Statistical methods included analysis of variance and logistic and linear regressions. Greater *T. gondii* antibody titers were found in suicide attempters than in non-suicide attempters (*p* = 0.004). No other hypothesis-driven or exploratory analyses yielded any significant result.

Inclusion criteria for patient groups was to meet DSM-IV criteria for Major Depressive Disorder or Bipolar I or II Disorder, and for controls was not to meet any criteria for axis I disorder (213). Suicide attempt history was obtained with The Columbia Suicide History Form (214).

Stored plasma samples were tested for IgG antibodies to *T. gondii* by solid phase enzyme immunoassay (as described previously) (215). Antibody levels were analyzed quantitatively (serointensity) and qualitatively (intensity of anti-*T. gondii* antibodies >10 international units). The laboratory technician was blind to diagnosis or caseness.

### Results

Using logistic regression, we found a significant association between serointensity level and suicide attempt [OR = 1.55 (1.14–2.12), *p* = 0.006]. The association of seropositivity with suicide attempt was not significant [OR = 1.62 (0.72–3.65)]. No association between any marker of *T. gondii* and number of suicide attempts was significant. There were no differences in *T. gondii* antibody levels between those with Major Depressive Disorder vs. Bipolar Disorder (*p* = 0.55), with vs. without a mood disorder (*p* = 0.22), or with vs. without psychotic symptoms (*p* = 0.34).

### Implications

This was the very first study connecting *T. gondii* and suicidal behavior. Limitations of this first study were that the information about suicide attempt predated, in some individuals, by a long time, the blood draw (a limitation shared by many future studies), and that the sample size did not allow for quantitative analyses and we could not adjust for socio-economic risk factors—important confounders. Because *T. gondii* seropositivity was not associated with a history of suicide attempt (due likely to smaller statistical power as a categorical vs. continuous variable), our report, while given the precedence over others in meta-analysis, has been reported as a negative study, as meta-analyses have generally not analyzed the serointensity (98, 105). While it is not known what specifically generates higher titers in specific individuals, the extent of cyst infection (mainly in the brain and muscle), the frequency and recency of reactivation, and a higher virulence of the infection are candidate factors. Certain patterns in results will be repeated in the future—the association with suicidal behavior appeared not to be mediated by associations with mental illness and reported number of suicide attempts did not relate significantly to *T. gondii* serology.

## The First Cohort Study of *T. gondii* and *Subsequent* Suicidal Behavior

The great majority of studies have usually related *T. gondii* seropositivity or serointensity to a psychiatric phenotype that had an onset prior to the results of the blood draw for the antibody test for *T. gondii*, confirming an infection with the parasite. It is thus possible that the presence of the psychiatric phenotype may have contributed to *T. gondii* infection. Indeed, decreases in self-care in mood and psychotic disorders, cognitive deficits, and the general impulsivity that accompanies presentations of a number of psychiatric syndromes may result in decreases in hand hygiene, washing of fruits and vegetables, cooking time of meat products, and the general decrease in socio-economic status may lead to general contamination with oocysts. For instance, some studies have attempted to relate *new onset* schizophrenia to *T. gondii*, in that way avoiding the long-term exposure to positive and negative symptoms and effects of medications. Nevertheless, having the blood test occur after the diagnosis maintains a good possibility of a “reverse causality,” i.e., that mental illness causes a greater risk for infection. Thus, having the *T. gondii* blood test performed before a psychiatric diagnosis in a large cohort may overcome the issue of reverse causality and provide data to support the hypothesis that *T. gondii* infection is causing the psychiatric phenotype, rather than the opposite. In this vein, a previous Danish cohort study on mothers showed that a high level of *T. gondii* IgG antibodies determined in neonatal blood spots was associated with significantly elevated risk of schizophrenia spectrum disorders (216). The same cohort study was used for a study on suicidal behavior, a collaborative effort between the Danish register research in Aarhus, Denmark (217, 218) (PI Mortensen) and University of Maryland School of Medicine (PI Postolache). This collaborative effort was initially supported by the National Institutes of Health for a project on allergens, allergy, and suicidal behavior, using data in Danish registers (NIMH R01- PI Postolache) (25, 78). We briefly present the study here.

### Methods

A neonatal screening for *T. gondii* (219) was the original aim of the cohort study involving pregnant women residing in 5 Denmark counties from 1992 to 1995 (representing 1/3 of the county). These women were given a choice to have their neonate screened for *T. gondii* antibodies shortly after delivery. The study included only the first delivery, if the mother gave birth several times during the study. Accurate linkage between national registers was enabled by a personal identification number listed in the Danish Civil Registration System (218).

In the grandparent study (219), 5 to 10 days after birth, a heel stick blood sample was obtained and stored on filter paper. Analysis of two 3.2-mm discs was done by enzyme immunoassay for *T. gondii* IgG antibodies (220). The level of antibodies was represented by the percentage of the optical density obtained from the World Health Organization international standard serum and the IgG level was calculated by obtaining the mean of the two results. An IgG level > 24 in a neonate was considered *T. gondii*-seropositive in the mother at the time of delivery. Because newborn children infected with the parasite do not produce *T. gondii*–specific IgG until about the age of 3 months (221) and since IgG crosses the placenta, the IgG antibodies were considered to be of maternal origin. Data were also available on first-trimester serum IgG level for $\frac{1}{4}$^th^ of the women in the study population. The mother-offspring *T. gondii* antibody titers were highly correlated (Spearman correlation = 0.76; *p* < 0.001).

ICD 8 and 10 codes for SSDV and suicide attempts were used, as described in Pedersen et al. (78). We used the date of death from suicide or the date of the first contact for self-directed violence (whichever came first) as the time of onset of self-directed violence. Excluding poisoning and “unspecified” as method, we also analyzed violent suicide attempts.

The Cox proportional hazards models (Cox regression) (222, 223) were used to estimate incidence rate ratios of self-directed violence, which were stated as relative risks. A comparison of estimated log minus-log survival curves was used to evaluate the proportional hazards assumption.

Adjustments included age at delivery, and, secondarily, history of self-directed violence (including suicide) in the parents of the mothers, time since first psychiatric contact, and psychiatric history. Mental illness history in parents and in women were treated as time-dependent variables. Analysis followed a categorical dichotomous model (seropositive and seronegative) and a ranked model with seropositive IgG levels divided into groups according to the 25th, 50th, 75th, and 90th percentiles. We also stratified, secondarily, for history of mental illness (present or not present). The likelihood ratio tests (222) were used to calculate *p*-values and 95% confidence intervals. The Danish Data Protection Agency approved the study.

### Results

Seropositivity, based on the bimodal distribution of IgG levels, was defined as an IgG level > 24, yielding a seropositivity rate at the time of delivery of 26.80% (95% CI, 26.33–27.28). As compared to *T. gondii*–seronegative mothers, seropositive mothers had a 1.53-fold (95% CI, 1.27–1.85; *p* < 0.001) significant relative risk of self-directed violence. When subdividing the seropositive values according to the 25th, 50th, 75th, and 90th percentiles, the risk of self-directed violence increased with the category based on *T. gondii* IgG levels. As such, women with an IgG level > 83 had a relative risk of 1.91 (95% CI, 1.25–2.79) relative to seronegative women. In violent attempts, the *T. gondii* effect was stronger—i.e., relative risk of a violent suicide attempt in seropositive women was 1.81 (95% CI, 1.13–2.84; *p* = 0.01), when compared with seronegative women. The sample size was not sufficient to analyze death by suicide as, in the cohort of 45,788 women, only 18 died by suicide during 604,844 person-years at risk. Yet, even if not significant given the small effect-size, the effect size seemed stronger for suicide as compared with SSDV or violent suicide attempt [2.05 (95% CI, 0.78–5.20; *p* = 0.14)].

### Implications

In this first cohort study, we and our collaborators and have identified, for the first time, an association between *T. gondii* IgG serology and self-directed violence at a subsequent time. This had the largest sample size, and at the time of the study, was the only study where exposure to *T. gondii* occurred prior to self-directed violence. In some meta-analyses, this study had not been included because it was falsely considered as not measuring suicide attempts, but non-suicidal self-directed violence only. In fact, the associations with violent suicide attempts were analyzed and have proven to be the strongest significant associations. We reported a predictive association between *T. gondii* IgG antibody titers soon after delivery and subsequent suicidal behavior. While approaching closer to causality because of the temporal sequence of exposure-outcome consistent with our hypothesis, and the dose-response effect illustrated by the serointensity category link with suicidal behavior, the study nevertheless is far from supporting a causal association. For example, it is conceivable that the results could be alternatively explained by people with latent or oligosymptomatic psychiatric disturbances with an increased risk for suicidal behavior having a higher risk of becoming *T. gondii* infected prior to mental health diagnosis. Washing the kitchen knives infrequently after preparation of raw meat prior to handling another food item, cleaning the cat litter box, incompletely washed fruits and vegetables, and consumption of raw or undercooked meat have been specifically reported to be factors elevating the risk of *T. gondii* infection in pregnant women (40, 224). Similarly, endophenotypes for suicidal behavior, such as impulsivity, represent risk factors for *T. gondii* seropositivity as well as future suicidal behavior, and, given the significant heritability of *T. gondii* (225), it may be possible that this increased impulsivity is brought about via genetic factors for exploratory or self-neglectful behavior. It is important to note that secondarily adjusting for history of mental illness and suicidal behavior in the parents (that would potentially reduce the effect of heritable elements mentioned above) had only a minor effect on the findings, making it unlikely that the results are mainly driven by heritable hidden variables related to pre-existing subclinical conditions, which would cause rather than be the consequence of *T. gondii* infection.

## In Schizophrenia: Tryptophan Degradation Pathway in Intersection With *T. gondii* Serology

As a result of a collaboration between T.T. Postolache and D. Rujescu from the University of Maryland School of Medicine and Munich University, respectively, as well as other collaborators, funded by the American Foundation for Suicide Prevention via a Distinguished Investigator Award, an analysis between history of suicide attempts and *T. gondii* serology was published in 2016 in the *Journal of Psychiatric Research* (95). As the kynurenine pathway of tryptophan degradation had been associated previously with chronic *T. gondii* infection (226, 227), history of suicide attempts (29, 202), and immune suppression (228–232), potentially affecting the capability of *T. gondii* to escape immune “pressure,” we investigated the effect of potential interactions between kynurenine (KYN), kynurenine-tryptophan ratio (KYN/TRP) and *T. gondii* serology on suicidal behavior.

By the time of this add-on project we had already reported the first association of *T. gondii* serology with suicide attempts in individuals with mood disorders (74) and then replicated it in a large sample of individuals with a diagnosis of schizophrenia (1,000 participants) (76), individuals with mood disorders in samples with mixed psychiatric disorders vs. healthy controls (75, 77), and in a prospective cohort study of Danish women (78). The association of *T. gondii* seropositivity with death by suicide had already been reported in women of post-menopausal age (72). Trait impulsivity and aggression, intermediate phenotypes for suicidal behavior (12), were found to be associated with *T. gondii* seropositivity in psychiatrically healthy individuals (114). The context that prompted this new analysis was the implication of inflammation in suicidal behavior (233). Specifically, levels of IL-6 and TNF were found to be increased in the plasma (160), and IL-6 levels were found to be increased in the CSF, of individuals with a history of suicide attempt relative to non-attempter controls (234). Moreover, individuals who died by suicide exhibited significant brain microgliosis (235). Likewise, increased IL-1β, IL-6, and TNF messenger RNA (mRNA) and protein were found in brain regions previously implicated histopathologically in suicidal behavior (236).

Several further connections seemed potentially meaningful for mechanistic considerations. The capacity of the inflammatory cascade, and specifically of interferon gamma (IFN-γ), to curtail the infection in immunocompetent individuals (226, 237–239) is in great part through starvation of *T. gondii* of tryptophan (TRP). Additionally, TRP is metabolized into KYN in a reaction catalyzed by IDO, that is induced by pro-inflammatory stimuli, and in a reaction catalyzed by tryptophan 2,3-dioxygenase (TDO), that is induced primarily by glucocorticoids. Kynurenic acid (KYNA) and QUIN are two important neuroactive metabolites of the KYN pathway (29). KYNA is an antagonist at NMDA glutamate receptors while QUIN is excitotoxic, as it stimulates NMDA receptors. Individuals with schizophrenia have been demonstrated to have higher seropositivity rates of *T. gondii* IgG (54, 61) and also to have elevated levels of KYN and KYNA in the brain and CSF (240, 241). A first study on the KYN/TRP system in individuals with suicide attempts found elevated KYN, but not lower TRP, to be positively associated with suicide attempt status (29). State dependent elevations of QUIN have been reported in persons with suicide attempts, a finding that was robust to adjustment for depression scores (202). Furthermore, reduced levels of picolinic acid (PIC), and PIC:QUIN ratio have been reported in suicide attempters (242). Thus, one key mechanism implicated in the association between *T. gondii* and suicidal behavior could be the parasite-induced and perpetuated low-grade inflammation leading to high QUIN and increased stimulation of glutamatergic receptors.

KYN and its metabolites could contribute to intermittent weakening of immune pressure directed on the parasite and maintaining it in its slow growing form, and potentially result in reactivation and formation of tachyzoites (fast growing forms). KYN induces down regulatory apoptosis of effector T cells, most notably T helper 1 (Th1) cells (228–232), downregulation of dendritic cell immunogenicity (243), and induction of regulatory T cell (Treg) differentiation (244) through the aryl hydrocarbon receptor (AHR), centrally involved in the generation of Treg (245). By analogy, expression of IDO is implicated in immune evasion of cancer (246, 247) with a direct role of KYN in reducing antitumor immune responses via AHR (248).

### Methods

Nine hundred and fifty participants with a DSM-IV SCID (213) diagnosis of schizophrenia were recruited from both outpatient and inpatient settings in Munich. The Positive and Negative Syndrome Scale for Schizophrenia was used to estimate symptom severity [PANSS (249)] and antipsychotic medication doses were expressed as chlorpromazine equivalents. Information regarding history of suicide attempts was obtained during clinical interviews conducted at the Department of Psychiatry, Ludwig Maximillian's University of Munich, Germany. Statistical analysis was based on logistic regressions.

### Results

Those with KYN in the upper 25th percentile had higher rates of suicide attempts than those with KYN in the lower 75th percentile, only in the *T. gondii*-positive participants, while in the *T. gondii* negatives no significant association was identified. There were no other differences in demographic and clinical variables between participants with KYN in the upper 25th percentile and those in the lower 75th percentile in the *T. gondii*-seropositive patients. In the entire group, KYN top quartile was not significantly associated with suicide attempts. Similarly, *T. gondii* seropositivity was associated with suicide attempt history, but only in those in the upper quartile for the plasma KYN level, while in those in the bottom three quartiles, the association was not significant. No significant association was found between a history of suicide attempts and plasma KYN as a continuous variable in the entire sample or *T. gondii*-based subgroups, or between a history of suicide attempts and *T. gondii* serointensity in the entire sample or KYN-based subgroups.

### Relevance

This is the first study that considered two recently uncovered molecular systems implicated by the Postolache group at the University of Maryland School of Medicine—in SSDV: *T. gondii* serology and the kynurenine pathway in interaction, rather than separately. Moreover, the finding of an association between *T. gondii* and history of suicidal behavior only occurred in those with high KYN. Similarly, high KYN was related to a history of suicide attempts only in those with seropositivity to *T. gondii*. The mediation, by KYN, of the *T. gondii* association (via pro-inflammatory signals in *T. gondii* positives) is highly unlikely, because KYN was not significantly associated (either as a continuous variable or its top quartile) with suicidal behavior in the entire sample.

The results are consistent with reciprocal interactions between the KYN pathway and infection. As one possibility, the results may be consistent with the immunosuppressant effect of KYN (228–232), potentially contributing to an intermittent reactivation of *T. gondii*. Without reactivation, potentially driven by a dominant vector of immunosuppression, the *T. gondii* seropositivity, *per se*, would not lead to an increased risk of suicidal behavior.

*T. gondii* has known effects on the brain that could be exacerbated by its reactivation and become synergistically enhanced by downstream neuroactive metabolites of the KYN pathway. For example, neuronal function could be directly affected by parasitic proteins (250). Specifically, in *T. gondii* tachyzoite-containing neurons, calcium signaling is impaired with most of the neurons being hyper-responsive to glutamate; QUIN, a metabolite of KYN, has additional additive or synergistic glutamatergic NMDA excitotoxicity. In the context of intracellular calcium, *T. gondii*-infected neurons did not return to normal after stimulation (250), leading to increased KYN (251), immunosuppression, and reactivation of *T. gondii*.

It is also conceivable that psychosocial challenges associated with living with a diagnosis of schizophrenia increase chronic psychological stress (252), and thus glucocorticoid stimulation and liver KYN production via TDO, engagement of the KYN metabolites, and downstream KYN-mediated secondary immunosuppression, thereby potentially leading to reactivation of the parasite.

Another possibility is that those with a history of *T. gondii* infection, and especially those with high KYN, may have chronic gastrointestinal inflammation that contributes to increased risk of suicidal behavior via mechanisms involving the gut microbiota and increased gut permeability, and perpetuation of inflammation with intermittent exacerbations, in particular, considering the schizophrenia diagnostic association with microbial dysbiosis (253, 254).

The study had several major limitations. The cross-sectional design invalidates causal inferences and the precedence of attempts before the blood draw further reduces causal claims. Additionally, the peripheral, rather than the CSF measurement of KYN, makes inferences about central interactions rather speculative. However, KYN freely crosses the blood-brain barrier in both directions. The study had several strengths: adjustment for several potential confounders in the statistical models given the relatively large sample size, the use of SCID in the diagnosis of schizophrenia, and diagnostic homogeneity by virtue of including only individuals with schizophrenia. While measuring CSF metabolites has more validity for mechanisms, measuring blood biomarkers, such as KYN, in peripheral blood increases the future possibility of its use in clinical settings.

In summary, it is only when both high KYN and seropositivity for *T. gondii* coexist in the same individual that the risk of suicidal behavior is elevated, in individuals with schizophrenia, at the very least. Predispositions, triggers, and perpetuating factors may be the driving forces of this finding. The presence of reciprocal interactions between KYN and *T. gondii* is a possibility to be investigated by future clinical and animal studies.

## *T. gondii*, Dopamine, and Endophenotypes of Suicidal Behavior

Increases in inflammatory markers have been associated with behavioral traits that have been proposed as endophenotypes of suicidal behavior, such as aggressive tendencies and behavior, anger, hostility, and impulsivity (12, 20, 191, 192, 255), as reported in animal studies (176–179) as well as human studies (180, 182–184, 256, 257). Experimental data point toward low-grade immune activation directly causing aggressive behavior (177) rather than representing pure epiphenomena, or non-specific consequences of stress or arousal.

Though considered harmless in immunocompetent hosts, “latent” *T. gondii* infection has been associated with subclinical personality traits (52, 258–263), as well as with personality disorders (264). We hypothesized that *T. gondii* serology is positively associated with intermediate phenotypes for suicidal behavior, especially traits such as impulsivity and aggression. We undertook the study in psychiatrically healthy adults (114), considering that in psychiatric conditions already linked with *T. gondii* seropositivity, associations between impulsivity and aggression could be, in part, consequences of psychiatric psychopathology we well as psychiatric treatment.

### Methods

IgG antibody seropositivity for *T. gondii* was analyzed in relationship to traits of impulsivity and aggression. For evaluating *T. gondii* specificity, two other latent neurotropic infections, cytomegalovirus (CMV) and herpes simplex virus 1 (HSV1) were also analyzed. The sample included 1,000 residents of the Munich metropolitan area [490 women, 510 men, mean age (SD): 53.6 (15.8)] with no Axis I or II conditions by SCID for DSM-IV. Trait impulsivity (Sensation-Seeking Scale-V [SSS-V]) and aggression scores were obtained from a German version of the Buss-Durkee Hostility Questionnaire, The Questionnaire for Measuring Factors of Aggression (FAF-Fragebogen zur Erfassung von Aggressivitätsfaktoren) (265, 266): FAF is composed of 76 items, of which 66 explore five components of aggressive behavior including FAF-Self-Aggression (11 items), FAF-Total Aggression (35 items) and its three component subscales: FAF-Spontaneous Aggression (19 items); FAF-Reactive Aggression (13 items); FAF-Irritability (13 items). Internal consistency of FAF was good as indicated by Cronbach's alpha values ranging from 0.61 to 0.79, and test-retest reliability was good as well (265).

The Disinhibition [SSS-V (DIS)] subscale of the Sensation Seeking Scale-V was used to measure impulsive sensation-seeking (267). The SSS-V was developed to evaluate differences in individuals' needs for stimulation and arousal (268, 269). Of the four SSS-V subscales, impulsive sensation was shown to have the strongest association with reckless behavior (270–272) and risky driving (273). The SSS-V (DIS) subscale scores have previously been used to predict repeated suicide attempts (274) and higher aggression (275), but mainly as a primary measure of impulsivity and to calculate an “impulsivity score.”

### Results

**Figure 5:**
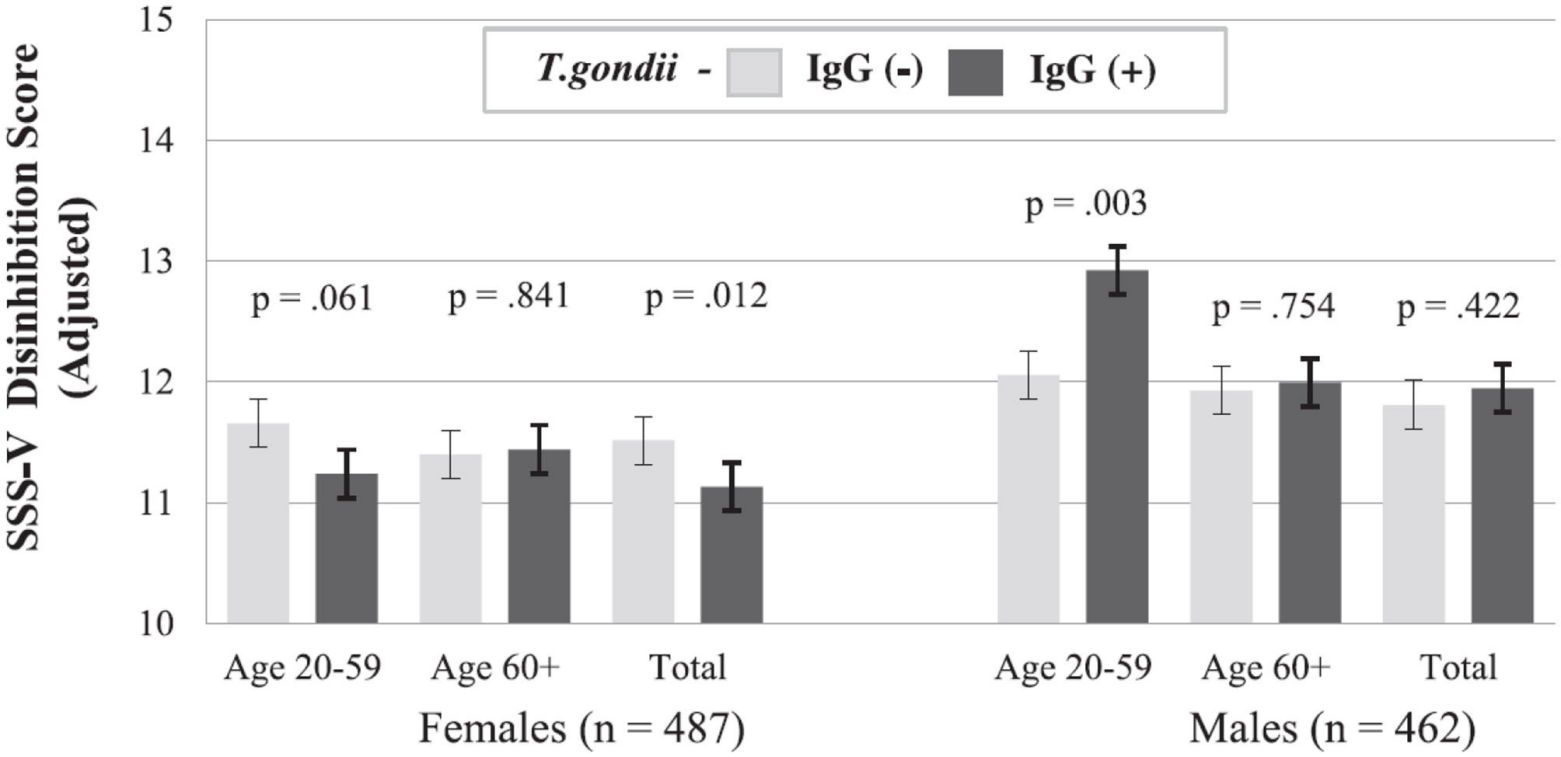
Trait Impulsivity (SSS-V Disinhibition) by Age, Sex, and *T. gondii* IgG Status (*N* = 949). Among younger men aged 20–59 years old, *T. gondii* seropositivity was significantly associated with higher impulsive sensation-seeking (SSS-V Disinhibition) (*p* < 0.01). The two age groups are separated by median age (60 years). *T. gondii* IgG (–), *T. gondii* IgG seronegative, *T. gondii* IgG+, *T. gondii* IgG seropositive, SSV = Sensation Seeking Scale-V [Reprinted with permission from (114); Copyright (2015); with permission from Elsevier; License # 4964160181012].

*T. gondii* seropositivity was associated with higher impulsive sensation-seeking (SSS-V Disinhibition) only among younger men (*p* < 0.01) aged 20–59 years old (median age = 60 years) (see Figure 5).

**Figure 6:**
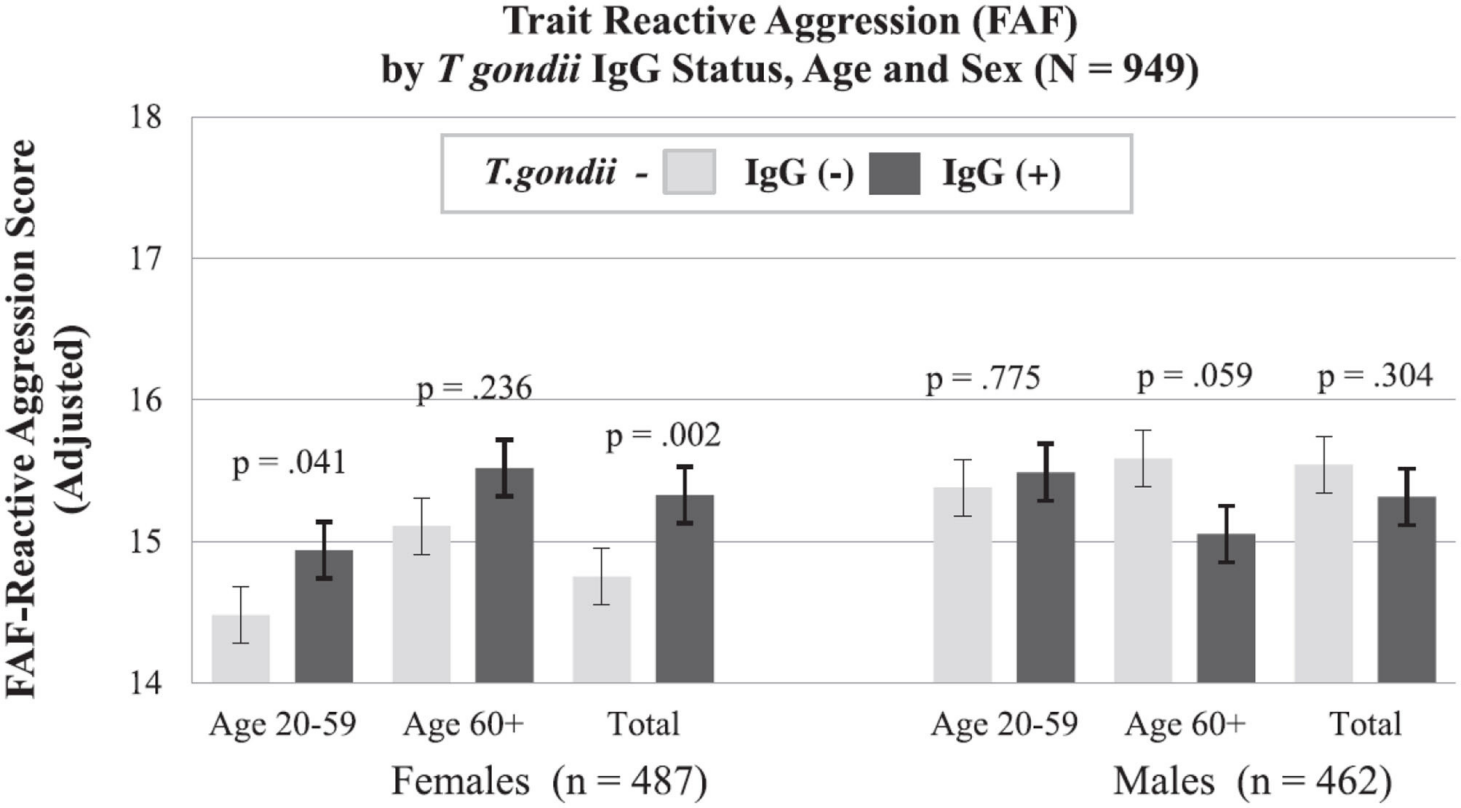
Trait Reactive Aggression (FAF) by Age, Sex, and *T. gondii* IgG Status (*N* = 949). Women, but not men, had a significant (*p* < 0.01) association between *T. gondii* IgG seropositivity and higher trait reactive aggression scores (determined by the FAF-Fragebogen zur Erfassung von Aggressivitätsfaktoren). *T. gondii* IgG (–), *T. gondii* IgG seronegative, *T. gondii* IgG+, *T. gondii* IgG seropositive. The two age groups are separated by median age (60 years). The overall reactive aggression scores are higher in men, but *T. gondii*-positive women have a similar level of reactive aggression with men. [Reprinted with permission from (114); Copyright (2015); with permission from Elsevier; License # 4964160181012].

Higher trait reactive aggression scores were associated with *T. gondii* IgG seropositivity in women only (see Figure 6). All associations with the other latent pathogens HSV1 and CMV were not significant, and adjustment for their serology did not reduce the significant *T. gondii* findings.

### Relevance

To our knowledge, using a study design that minimizes confounding effects of mental illness, trait aggression and impulsivity had never been studied in relation to latent Toxoplasmosis.

The sex differences in our findings were not surprising, given previous sex-specific associations between personality and *T. gondii* (258, 260, 261, 263, 276), and may represent expressions of different vulnerabilities to the parasite, immune changes in Toxoplasmosis, or propensity for behavioral dysregulation. However, since, experimentally, sex-specific behaviors (263, 277, 278), secretion of gonadal steroids (279), and sex-specific neurotransmitter changes (280) occur in rodents infected with *T. gondii*, sex-specific effects of the parasite represent a plausible explanation for the observed association.

These results are consistent with experimental studies. *T. gondii* infection increases novelty-seeking in rodents and non-specifically reduces anxiety-like behaviors (41–45), with the localization of *T. gondii* predominantly in brain regions involved in fear modulation and / or induction, such as the prefrontal cortex and amygdala (193, 194). Reduced fear- and anxiety-like behavior may be the result of dendritic retraction in the basolateral amygdala (151). In terms of neurotransmitters underlying the connection between *T. gondii* impulsivity and aggression, dopaminergic signaling represents a top candidate mechanism, as dopamine production was demonstrated to be elevated with *T. gondii* infection (281). The genome of *T. gondii* has two genes coding for tyrosine hydroxylase (195), and PC12 cells infected with *T. gondii* release higher levels of dopamine in response to stimulation than non-infected cells (48). Furthermore, in acutely *T. gondii*-infected rodents, dopamine metabolism appears somewhat slowed down while brain dopamine levels are elevated (282, 283). Furthermore, in *T. gondii*-infected mice mRNA expression for three genes involved in dopamine signaling MAO-A, Drd1, and Drd5, encoding monoamine oxidase A and the D1 and D5 dopamine receptors, respectively, is decreased (283).

The neurobiology of impulsivity draws heavily from dopaminergic neurophysiology (284–295). Inconsistencies occur, with literature suggesting a negative (288, 292, 296–299), as well as a positive association (300–302). This heterogeneity may be a result of complexity of neural circuits involved in dopamine neurotransmission, as well as the topographic specificity of dopamine receptor subtypes (288, 292, 296–299). Moreover, genetic polymorphisms in enzymes associated with dopamine transporter activity, dopamine receptors, and dopamine metabolic pathways have been associated with impulsivity (291, 299, 303–305). Furthermore, genetic polymorphisms in enzymes associated with dopamine receptors and dopamine transporter activity, as well as dopamine metabolic pathways, may contribute to heterogeneous associations with impulsivity (291, 299, 303–305).

Impulsivity in *T. gondii* infection could be modulated by dopaminergic neurotransmission altered by the dopamine producing and altering parasite. MicroRNA-132 is substantially upregulated by all three-prototype *T. gondii* strains (283). Moreover, in the same study, upregulation of microRNA-132 was found to be associated with decreasing the metabolizing enzyme (i.e., monoamine oxidase A), changes in dopamine receptor signaling, and decreasing expression of D1-like dopamine receptors, a dopamine receptor involved in the negative feedback regulation of dopamine release in the brain (306), resulting in higher levels of dopamine. Indeed, in striatal tissue of mice infected with *T. gondii*, dopamine levels are elevated by 38% and microRNA-132 is upregulated (283). Additionally, elevated levels of homovanillic acid, a dopamine metabolite, and increased synthesis of dopamine have been found in dopaminergic neurons infected with *T. gondii* (48, 281, 307). As a higher acoustic startle response magnitude has been associated with increased dopamine production (308, 309), an increased startle response among *T. gondii*-infected participants (310) has been attributed to increased dopamine production in *T. gondii* positives. The prevention of *T. gondii*-induced behavioral alterations in animals with anti-dopaminergic agents further supports a *T. gondii*–dopamine connection (311, 312). Consistently, dopamine D2-receptor agonists reduce response impulsivity in humans (292) and, experimentally, in rodents (288). Conversely, premature responding in highly impulsive rats is enhanced by administration of the dopamine D2/D3 receptor antagonist nafadotride into the accumbens shell in rats (313). Moreover, low dopamine D2-like receptor mRNA expression in the mesolimbic pathway was identified in highly impulsive rats (314). Additionally, a greater response impulsivity in a motor impulsivity animal model is manifested in rats that have lower dopamine D2/D3 receptor availability in the nucleus accumbens (285). In human participants, inhibition-related functional magnetic resonance imaging (fMRI) activation in frontostriatal circuitry positively correlates with dopamine D2/D3 receptor availability and negatively correlates with impulsivity (315). Additionally, a negative association with levels of impulsive aggression have been reported with CSF levels of homovanillic acid, the main metabolite of dopamine (316). There are findings that suggest a positive, rather than a negative, association between dopaminergic function and impulsivity (300–302). Yet, all studies support a robust effect of dopaminergic modulation of impulsivity and suggest that *T. gondii*, through cellular and biochemical correlates consisting of reductions in neuronal spine density and dopamine levels, as demonstrated in the nucleus accumbens core (317), can modify impulsivity—a major risk factor and intermediate phenotype of suicidal behavior.

Variations in gonadal steroid levels associated with *T. gondii* infection in humans, implicated in aggression and self-directed violence, may contribute to our reported sex and age differences (283, 318–322). An indirect support for the hormonal mechanism of sex-age group differences is that FAF-Self-Aggression scores among *T. gondii*-positive women switches from lower to higher around the average menopause age of German women (323, 324). Animal studies also support potential sex differences comparing monoamine neurotransmission in male vs. female rats in acute and chronic infection demonstrating sex-differences (280). Gonadal steroids are increased in *T. gondii*-infected rats, but not mice, and play a role in behavioral changes induced by chronic Toxoplasmosis in male rats (278, 279), but not female rats. In humans associations between suicidal behavior and blood levels of gonadal steroids have been reported in both women (324) and men (325–327).

**Figure 7:**
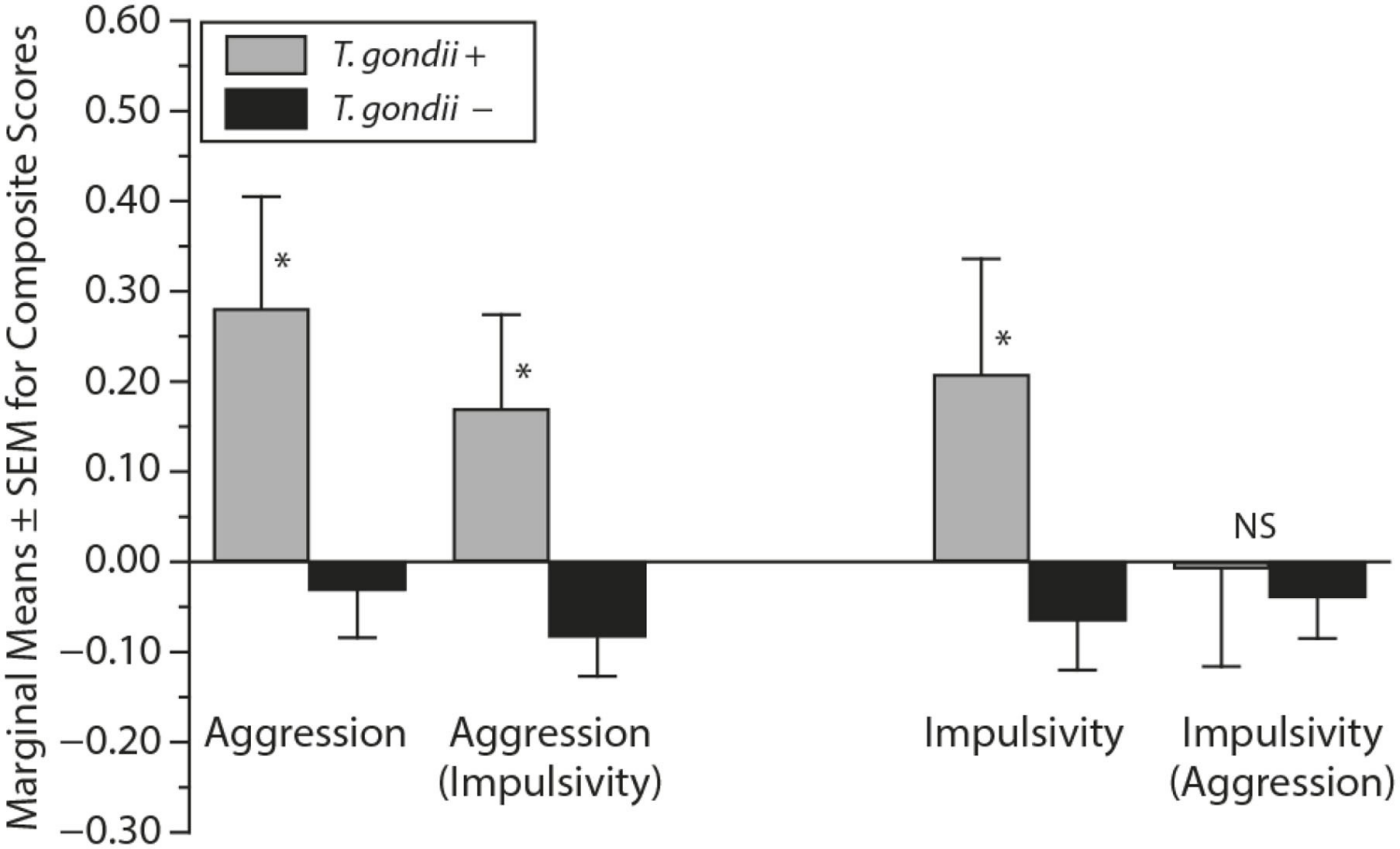
Composite Impulsivity and Aggression (age as covariate) in *T. gondii* seronegative (–) and seropositive (+) participants with history of Intermittent Explosive Disorder. Impulsivity (aggression) refers to Composite Impulsivity scores with Composite Aggression scores as a covariate; aggression (impulsivity) refers to Composite Aggression scores with Composite Impulsivity scores as a covariate. NS, not significant; *p ≤ 0.05. Significantly elevated Aggression and Impulsivity scores in *T. gondii* positives. Reciprocally adjusting impulsivity and aggression for each other yields a significant association with *T. gondii* only for Aggression [Reprinted with permission from (117); Copyright 2016, Physicians Postgraduate Press. Reprinted by permission].

**Figure 8:**
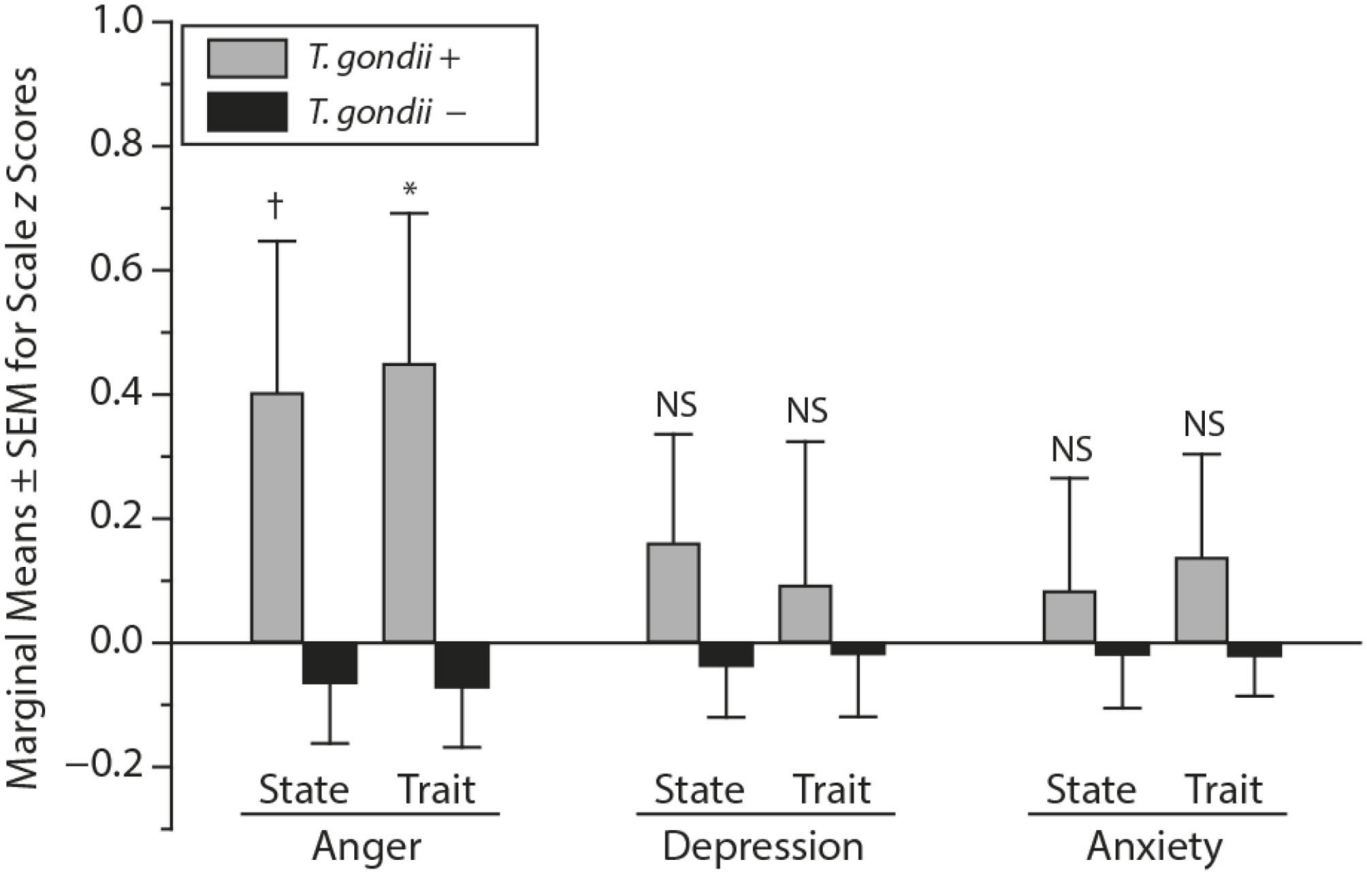
State and Trait Anxiety, Anger, and Depression (*z*) scores (age as covariate) in *T. gondii* seronegative (–) and seropositive (+) participants. *z* scores were used to place all symptom measures on the same scale. NS, not significant; ^*†*^*p* < 0.10; *p ≤ 0.05. While State and Trait Anxiety and Depression were not significantly different between *T. gondii*-positive and *T. gondii*-negative participants with Intermittent Explosive Disorder, the State and Trait Anger is significantly higher in *T. gondii*-positive participants relative to *T. gondii*-negative participants [Reprinted with permission from (117); Copyright 2016, Physicians Postgraduate Press. Reprinted by permission].

In addition to being a cross-sectional study, there were limitations worth acknowledging. No history of actual episodes of impulsive behavior or aggression were acquired. No direct objective measures of behavioral aggression or impulsivity were obtained and neuropsychological testing of impulsivity did not take place. Moreover, aggressive and impulsive traits could modify the likelihood of exposure to *T. gondii* and, thus, of *T. gondii* infection. There may be residual confounding by unmeasured socioeconomic factors, as the proxy use of educational attainment could be of only limited value. Finally, including only healthy individuals (actually super healthy—given exclusion of those with family history of mental illness) limits the generalizability of the study. Yet, we believe that lower generalizability is a fair price to pay, considering the uncovering of personality traits linked with *T. gondii* in a sex- and age-specific fashion, even when the confounding of underlying mental illness and substance abuse is eliminated by design. Moreover, we were able to replicate the association between *T. gondii* and impulsivity/aggression traits in a clinical population with extreme impulsive-aggression, specifically Intermittent Explosive Disorder (IED) (117)—(see Figures 7, 8).

## Endophenotypes of Suicidal Behavior (Impulsivity, Aggression) And *T. gondii* Serology—the Moderating Role of Age, Gender, and Plasma Phenylalanine:Tyrosine Ratio

This project (115) representing a follow-up on a previous study (114) that uncovered that *T. gondii* seropositivity is positively associated with impulsive sensation seeking in younger men. The study was based on the concepts that impulsivity is regulated by dopaminergic and serotonergic signaling, and, because *T. gondii* is known to directly affect dopaminergic signaling and tryptophan degradation pathways via immune activation, blood levels of precursors of serotonin and dopamine may change the association between *T. gondii* and impulsivity, and thus risk of suicide. In addition to dopamine, serotonin is another key neurotransmitter involved in impulsivity regulation and dysregulation (328–331). Activation of the KYN pathway of TRP metabolism, leading to diversion of TRP from production of serotonin (226), results in reduced serotonin production and an elevation of impulsivity and impulsive aggression—intermediate phenotypes of suicidal behavior. Measures of serotonin, norepinephrine, and dopamine have been found to be altered in suicide brains (332).

The first step in the synthesis of dopamine is the conversion of the amino acid Phe to Tyr, catalyzed by the enzyme Phe hydroxylase (PAH). It is followed by a two-step process, with Tyr being converted into dopamine via Tyr hydroxylase (the rate-limiting enzyme in catecholamine biosynthesis) and L-type amino acid decarboxylase (333, 334). The Phe:Tyr ratio is an inverse estimate of PAH activity (335), and is higher in pro-inflammatory conditions, including trauma and sepsis (336), human immunodeficiency virus infection (337), cancer (338), as well as psychiatric conditions such as depression (339, 340) and schizophrenia (341–343). The Th1 immunity leads to depletion of (6R)-L-erythro-5,6,7,8-tetrahydrobiopterin (BH4), an essential cofactor of PAH (335, 344). Thus, the dysfunction of PAH results in a high Phe:Tyr ratio (340, 345), as a consequence of Th1 activation (335, 344), which, in turn, has been presented above as an important mechanism (346) to keep *T. gondii* in check by applying immune pressure to prevent its reactivation. This could lead to a causal association between *T. gondii* infection and high Phe:Tyr ratio. At the same time, the dopaminergic alterations potentially induced by *T. gondii* may be compounded by the low availability of Tyr through inflammation-induced PAH dysfunction.

### Methods

In 950 psychiatrically healthy participants as described above and in Cook et al. (114), *T. gondii* IgG seropositivity was related to trait impulsivity scores (calculated as explained in detail in different reports) (114, 115) in interaction with categorized levels (top 25% vs. bottom 75%) of Phe, Tyr, Kyn, and Trp measured by high performance liquid chromatography (HPLC), as described elsewhere (343, 347). Phe:Tyr ratio and the Kyn:Trp ratio were calculated, and age and gender were used for adjustment in analysis of covariance (ANCOVA) with impulsivity scores as a dependent variable. Participants were stratified into two categories based on their age (i.e., older and younger), based on a median split (60 years old).

### Results

**Figure 9:**
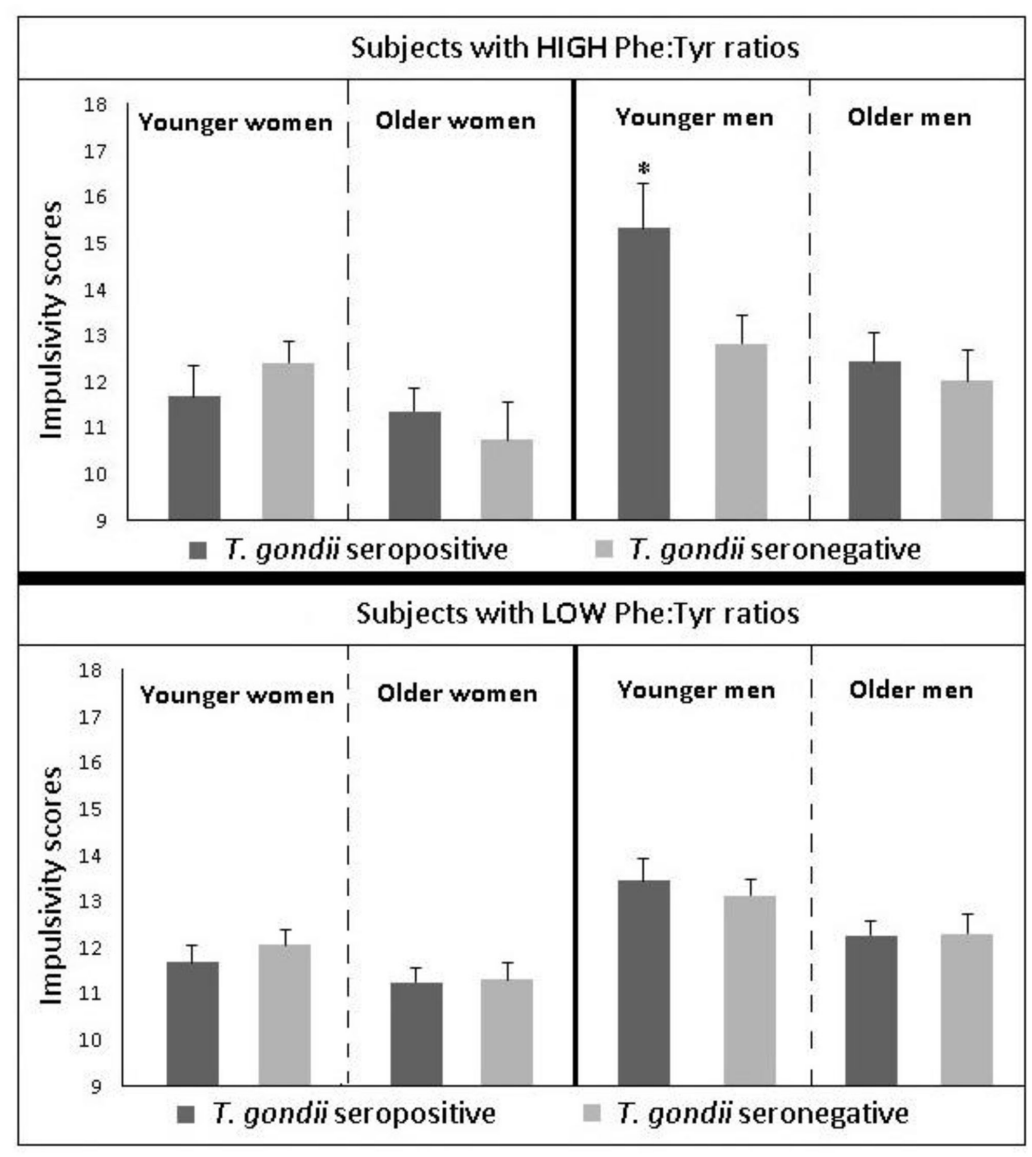
Endophenotypes for suicidal behavior. Comparisons of impulsivity scores between *T. gondii* IgG-seronegative vs. *T. gondii* IgG-seropositive individuals and high vs. low phenylalanine/ tyrosine ratio (Phe:Tyr) stratified by gender and groups. Scoring significantly higher on impulsivity scores than other groups required four coexistent criteria, i.e., for a participant to be male, young, *T. gondii*-positive, and in the upper quartile of Phe:Tyr. Impulsivity scores in seronegative vs. seropositive participants stratified by Phe:Tyr categories, gender, and age. Disinhibition subscale of the Sensation Seeking Scale-V [SSS-V (DIS)] was used to obtain impulsivity scores, which are represented as standard errors (SEs) and least square means (adjusted for age in the age category and stratified by categorical variable). The following strata were included: older women (aged ≥ 60 years), older men (aged ≥ 60 years), younger women (aged 20–59 years), and younger men (aged 20–59 years). If the Phe:Tyr ratio was in the lower 75th percentile, it was considered LOW and if the ratio was in top 25th percentile, it was considered HIGH. Statistically significant interactions were uncovered between *T. gondii* seropositivity, gender, age category, and Phe:Tyr ratio upon performing ANCOVA analysis of impulsivity scores [*F*_(1, 896)_ = 7.772, *p* = 0.007]. A significant interaction between Phe:Tyr ratio and *T. gondii* seropositivity status was present in younger men [*F*_(1, 173)_ = 10.635, *p* = 0.001], but it was not significant in other strata {i.e., older women [*F*_(1, 84)_ = 1.868, *p* = 0.173), younger women [*F*_(1, 280)_ = 0.516, *p* = 0.473], older men [*F*_(1, 256)_ = 0.593, *p* = 0.442]}. *Upon performing Tukey's Honestly Significant Difference Test, the impulsivity scores were significantly higher in younger men who had HIGH Phe:Tyr ratios and were also *T. gondii*-seropositive, as compared to all other subgroups (*p* < 0.01 for all). Additional significant differences within the subgroups were not present [Reprinted with permission from (115); Copyright (2018); with permission from Elsevier; License # 4964311353323].

An interaction was identified between seropositivity status for *T. gondii* and Phe:Tyr ratio category [*F*_(1, 173)_ = 10.635, *p* = 0.001] that was statistically significant only in younger men. A *post hoc* Tukey's Honestly-Significant-Difference test indicated that only in younger men (aged 20 to 59 years), who also had high Phe:Tyr ratios (i.e., ratios that fell into the top quartile), were statistically significant differences in the impulsivity scores between *T. gondii* seropositive and seronegative individuals identified. In all other groups, there were no statistically significant findings (see Figure 9).

Phe:Tyr could be just a marker of severity through immune-activation responses, and that may explain why the Phe:Tyr ratio alone does not significantly associate with impulsivity (the ratio is not a mediator, only a marker of severity) and why the *T. gondii* serology alone does not relate to impulsivity (only a more severe infection—extensive, virulent, and neurotropic, leading to a stronger immune response—would be sufficient to induce higher impulsivity).

**Figure 10:**
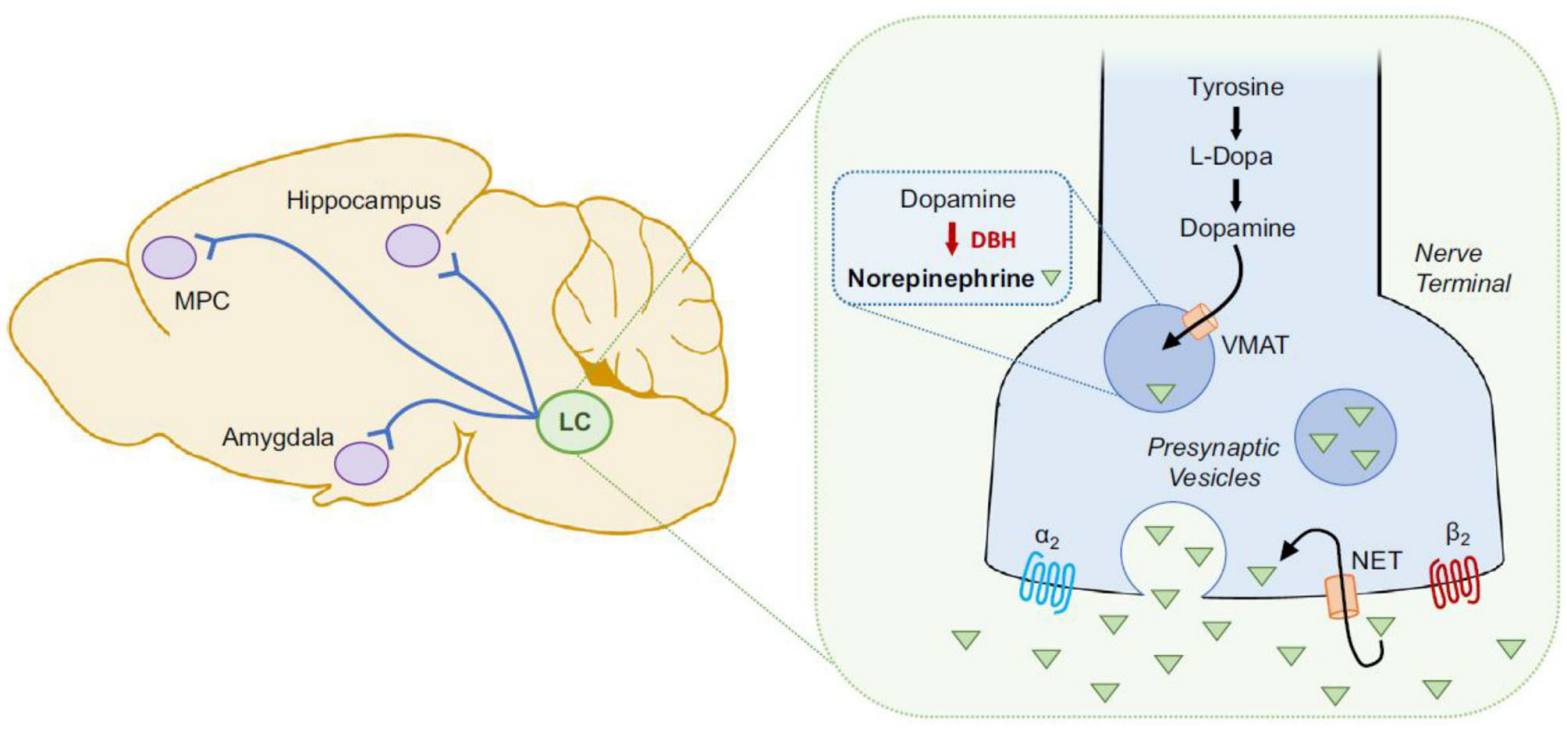
Graphic illustration of norepinephrine (NE) synthesis in the brain of a rodent. Synthesis of NE occurs from tyrosine through the following steps: (a) tyrosine hydroxylase converts tyrosine to levodopa or l-3,4-dihydroxyphenylalanine (L-DOPA); (b) L-type amino acid decarboxylase converts L-DOPA to dopamine; (c) vesicular monoamine transporter (VMAT) transports dopamine into the presynaptic vesicles; and (d) dopamine-β-hydroxylase (DBH) converts dopamine into NE. NE binding to β- and α- adrenergic receptors takes place after its release from the presynaptic vesicles into the synapse, from where norepinephrine transporter (NET) mediates its reuptake. Locus coeruleus (LC) houses the majority of brainstem noradrenergic neurons and it projects to several regions in the brain, including the amygdala, hippocampus and medial prefrontal cortex (MPC) [Reprinted from (363); Copyright (2020); with permission from Elsevier; License # 4986231317003].

**Figure 11:**
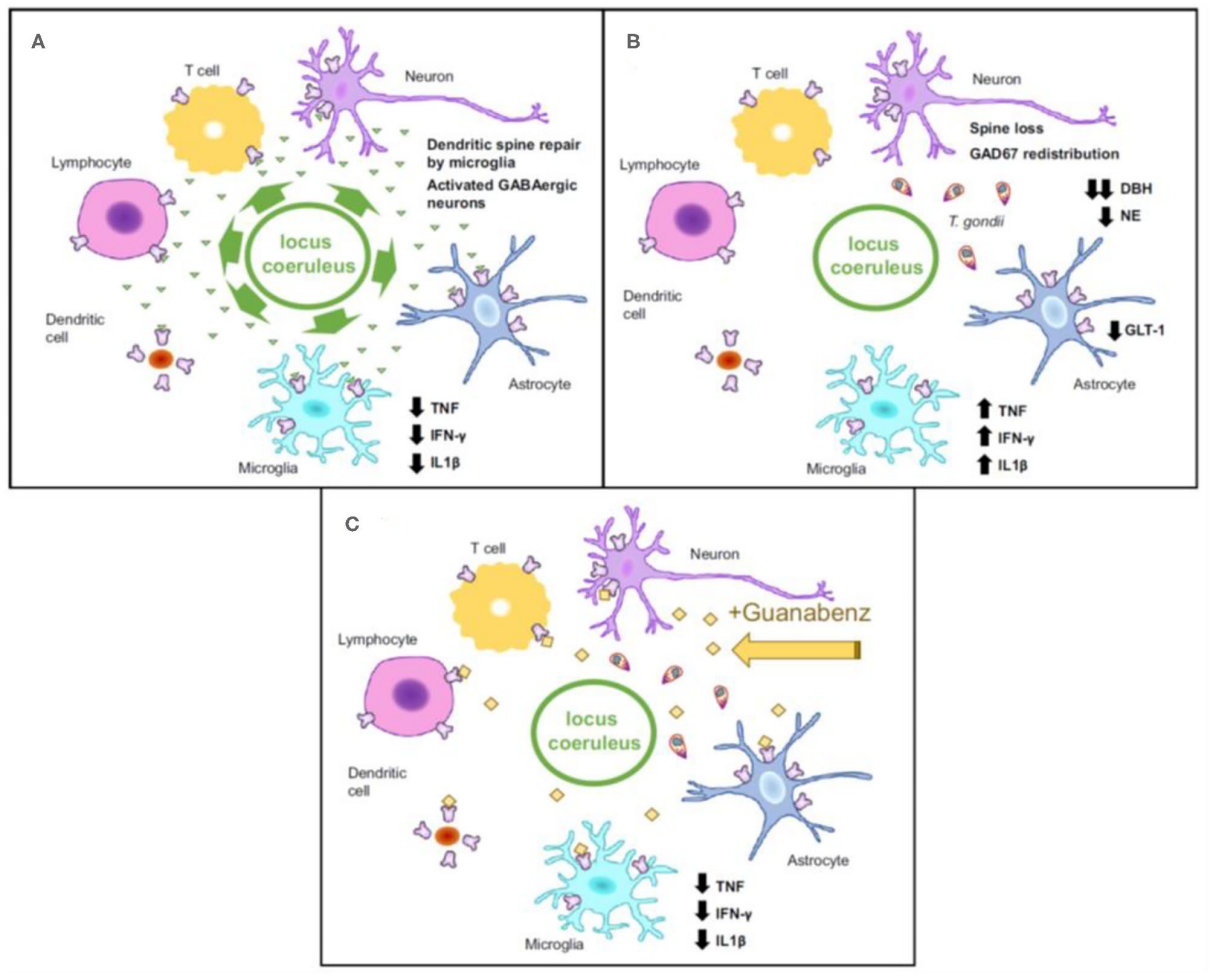
Schematic illustrating the regulation of inflammatory response by noradrenergic neurotransmission during an infection in the rodent brain. **(A)** Norepinephrine (NE, green triangles) is released from locus coeruleus noradrenergic neurons that are functioning normally. NE binds to adrenergic receptors located on immune cells, astrocytes, neurons, and microglia. Microglia and GABAergic neurons are activated by NE, cells that help with downregulation of pro-inflammatory cytokines and neuronal dendrite repair. **(B)** In rodents, neurons have been reported to undergo several changes in response to chronic *T. gondii* infection, including severe reduction in NE and dopamine-β-hydroxylase (DBH), within noradrenergic neurons, redistribution of GAD67 (an enzyme involved in GABA synthesis), and loss of dendritic spines. Additionally, reduced glutamate transporter Glt-1 has been observed in astrocytes of rodents chronically infected with *T. gondii*. Elevation in the levels of inflammatory cytokines, including interleukin (IL)-1β, tumor necrosis factor (TNF) and interferon (IFN)-γ also occurs. The model from Laing et al. states that since noradrenergic signaling is suppressed during chronic *T. gondii* infection, the brakes on inflammation are lifted leading to increased cytokine concentrations. The behavioral alterations seen in rodents chronically infected with *T. gondii* could be partly explained by these changes. **(C)** Laing et al. suggest that the elevated pro-inflammatory cytokines can be suppressed and the lack of NE can be compensated for (at least partially) by treating the *T. gondii*-infected rodents with Guanabenz (a noradrenergic agonist), which has been linked with reversal of *T. gondii* infection-induced hyperlocomotion in rodents) [Reprinted from (363); Copyright (2020); with permission from Elsevier; License # 4986231317003].

Furthermore, somewhat arguing against an immune hypothesis, in another study on impulsivity and aggression in a clinical population (i.e., individuals with IED), IL-6 did not appear to mediate our uncovered significant association between IED diagnosis/ trait impulsivity in IED and *T. gondii* seropositivity (117). However, we cannot rule out the role of other cytokines not analyzed in that study, including IFN-γ and TNF, which could specifically induce PAH and act to restrain *T. gondii* (226, 237–239). It is also possible that changes in IL-6 levels are not seen in peripheral blood circulation as the pro-inflammatory processes keeping *T. gondii* from reactivation are limited to the CNS. The possibility of alternate genetic, developmental, clinical, and environmental enzymatic predispositions toward inhibition of PAH activity in response to pro-inflammatory stimuli cannot be ruled out. Furthermore, it is possible that lower norepinephrine synthesis, in the context of reduced Tyr (see Figure 10) and infection, may lead to increased hopelessness, and then additionally, contribute to a reduced immune-modulatory activity of the noradrenergic system (see Figure 11). Indeed, several studies have implicated the noradrenergic system in suicidal behavior (332, 348, 349).

### Relevance

Treatment trials focused on reducing the elevated risk of suicide attempt in individuals seropositive for *T. gondii* could focus on impulsivity as a mediator, and, for that purpose, the study could specifically only include younger seropositive males with elevated Phe:Tyr ratios. Second, if the insufficient tyrosine availability combined with an increase in dopamine secretion in young *T. gondii*-seropositive males could have a mediating role, elevating Tyr levels in those with high Phe:Tyr ratios could, potentially, limit the increased risk of suicidal behavior due to elevated impulsivity in this specific group. Thus, simply restoring Tyr (widely available as a nutritional supplement and relatively safe) instead of a more complicated and potentially risky intervention, i.e., “sterilizing” *T. gondii* and thus preventing a chronic latent infection, may achieve a desired level of therapeutic benefit, and deserves testing in small and, ultimately, larger clinical trials.

## Hopelessness and *T. gondii* Serointensity in Old Order Amish

An association between chronic infection with *T. gondii* and depression has been reported in various study populations, such as pregnant women (66), psychiatric patients (67), and women Veterans (34); although this association has not been confirmed in other studies (53, 68–71). Inconsistencies in these findings could potentially be a result of heterogeneity in the study samples, which may include distinct associations between individual symptoms of depression and chronic Toxoplasmosis, co-morbid substance use disorders, or lifestyle variations among study subjects. To minimize these confounding effects, the Postolache team at the University of Maryland conducted a cross-sectional study in the Old Order Amish (OOA) of Lancaster, Pennsylvania (155), who, as compared to the general US population, have comparatively higher prevalence of *T. gondii* infection, lower substance use prevalence, and are rather homogeneous in terms of cultural practices, socioeconomic status and lifestyles (40, 350, 351). In this study, potential associations between *T. gondii* IgG serology and two predominant state markers for suicidal behavior, both translatable to animal models (anhedonia and dysphoria/hopelessness), were explored.

### Methods

Details about the study methods are described elsewhere (155). In summary, the study sample included OOA (*N* = 306) [mean age = 46.1 ± 16.7 years; 62.4% women]. Patient Health Questionnaires (PHQ-2 and PHQ-9) were used to ascertain cardinal symptoms of depression (anhedonia and dysphoria/hopelessness) and ELISA was used to measure *T. gondii* IgG titers. Multivariable linear methods were used to analyze the relationship between *T. gondii* IgG antibodies (serointensity and seropositivity) and various combinations of time-dependent cardinal symptoms of depression (i.e., current/past/ever dysphoria/hopelessness and anhedonia scores), while adjusting for gender and age. Participants were described as having “current” symptoms of depression if they experienced these symptoms in the last month.

### Results

*T. gondii* IgG serointensity had a significant positive association with current combined anhedonia and dysphoria/hopelessness [OR = 1.34 (95% CI: 1.01–1.79); *p* = 0.043], as well as current dysphoria/hopelessness [OR = 1.26 (95% CI: 1.01–1.58); *p* = 0.045]. However, the association between *T. gondii* serointensity and current anhedonia (in isolation) was not significant. The relationships between *T. gondii* serointensity and current predominant anhedonia and dysphoria were also non-significant when either (but not both) of them were present in the study subjects in the past month (i.e., current dysphoria/hopelessness *or* anhedonia). Additionally, significance was not reached when associations between *T. gondii* serointensity and past/ever mood phenotypes were analyzed. With regards to *T. gondii* seropositivity, its association with current combined dysphoria/hopelessness and anhedonia [OR = 2.99 (95% CI: 0.97–9.15); *p* = 0.056] and current dysphoria/hopelessness [OR = 2.31 (95% CI: 0.97–5.50); *p* = 0.058] revealed a statistical trend/low-grade significance.

### Relevance

These results point toward associations between *T. gondii* IgG serointensity and dysphoria/hopelessness. In patients with depression, dysphoria/hopelessness is an accepted risk factor for suicide (352, 353). Trait- and state-related hopelessness have both been reported to be present in an equivalent quantifiable degree in patients with depressive disorders (354). Trait hopelessness has been specifically linked to personality traits, including low Extraversion (355) and high Neuroticism (354, 356). Notably, trait hopelessness has shown a stronger risk with suicidal behavior than state hopelessness (357). Our study generates the new hypothesis that chronic *T. gondii* infection may elevate risk of suicide via increasing propensity to trait hopelessness, a hypothesis that warrants further research.

The association with serointensity, rather than with seropositivity, may suggest that it is not the infection *per se*, but its extent, virulence, and recency that are predictively linked with hopelessness, and thus an increased suicide risk.

At the molecular level, *T. gondii* infection-induced siphoning of tryptophan (a precursor for serotonin) (358) toward the kynurenine pathway, instead of the serotonin pathway (227), could explain the associations that we had uncovered in this study. IFN-γ is released as a response to *T. gondii* infection, which in turn upregulates IDO—the rate-limiting enzyme that directs the metabolism of tryptophan to kynurenine metabolites in the brain (359). This molecular shift is supposed to be protective for the host, as it leads to a relative deficiency of tryptophan, an amino acid that is essential for *T. gondii*'s growth (227). However, it leads to the production of more kynurenine and other metabolites of the kynurenine pathway, and results in a relative deficiency of serotonin in the brain—well-known to be associated with depression (360). Moreover, higher CSF levels of QUIN (metabolite of kynurenine pathway) (202, 242) and plasma levels of KYN itself (29) have been reported to be associated with a history of suicide attempts in individuals with depressive disorders (361). Additionally, chronic *T. gondii* infection may inhibit noradrenergic signaling, which, in addition to contributing directly to alterations in mood states, may also lead to a lower tonic inhibition of excessive neuroinflammation (see Figure 11), ultimately resulting in neuronal damage, changes in brain connectivity and cognitive deficits, thus contributing to the risk for suicidal behavior (122, 362, 363).

Some of the limitations of this study include a cross-sectional design, not using standardized structured instruments and collateral information to diagnose depression, and potential underreporting of symptoms of depression due to reporting/recall bias or cultural factors. The strengths included a much greater *T. gondii* seroprevalence in the OOA than in the US population (40, 350), allowing us to conduct multivariable analyses, and less heterogeneity (occupation, meal preparation, food consumption, hygienic practices, and social supports, as well as very limited use of tobacco, alcohol, and other substances) (350, 351).

Long-term future longitudinal and interventional studies on individuals who have report dysphoria/hopelessness (with/without anhedonia) potentially related to a chronic infection with *T. gondii* could test the might from certain targeted approaches to treatment involving psychotherapeutic and pharmacological interventions. In depressed patients, cognitive behavioral therapy has shown promising results in reducing dysphoria/hopelessness (364, 365). Additionally, anti-inflammatory interventions for seropositive individuals who have increased inflammatory markers in their blood, as advocated recently (366–368), primary prevention strategies to reduce targeted gender-specific risk factors for infection (40), and anti-parasitic medications in those with evidence of high frequency of parasite reactivation with temporally adjacent exacerbation of cardinal depression symptoms, could all help mitigate a possible mediator role of the *T. gondii*-suicide connection—i.e., dysphoria/hopelessness.

## Overall Implications, Limitations, and Conclusion

Three meta-analyses confirmed our initial reported associations between *T. gondii* and suicidal behavior. Furthermore, serointensity-response associations, linked with endophenotypes of suicidal behavior, and PAF analyses, suggest a potential sizable benefit of (1) preventing *T. gondii* infection; (2) treating the infection once it has occurred; or (3) intervening on molecular systems that interact with the parasite and may contribute to suicide risk. Together, these strategies have the potential to mitigate the potential pro-suicidal effect of *T. gondii* infection. Candidate molecular moderators may include elevated plasma kynurenine for pro-suicidal effects of *T. gondii* in schizophrenia and high plasma Phe:Tyr ratio for impulsivity-elevating effects of *T. gondii* in young males with no psychiatric history.

Our review, as well as the field in general, have substantial limitations. The limitations of our current review include its non-systematic nature and the presentation of results of different studies in the way it was reported originally in each study, rather than integrating it. Even for the results of the three meta-analyses, we allowed original differences to be apparent, rather than homogenizing or meta-analyzing the three studies. We thought that three independent meta-analyses that were concordant in confirming the *T. gondii*–suicidal behavior associations, despite differences in approach and especially study selection, represent stronger arguments to validate our initial observations, rather than a synthesized macro-analysis by our team, which would be certainly not immune to self–confirmatory bias. Limitations of this field include: (a) the absence of a clear documentation of *T. gondii* CNS localization in chronic Toxoplasmosis in immunocompetent humans [our team is currently completing such a report using high resolution structural MRI associations with IgG anti *T. gondii* oocyst, i.e., infection occurring directly through oocyst, rather than tissue cyst (manuscript in preparation)]; (b) in contrast to European countries (72), a lack of a correlation between national suicide rates and *T. gondii* seropositivity worldwide. These suggest that interactive factors related to *T. gondii* (serotype, transmission modality) and host (genetics, suicide risk and protective factors of bio, psycho, socio, cultural and economic nature) may have impactful moderating effects; and (c) causality has not been demonstrated; “hidden variables” and reverse causality are possible. Future interventional randomized studies will be necessary to confirm our causal inferences, and, if so, to seek to establish in suicidology, a first etiology-based treatment, and hopefully, primary prevention.

Theoretically, while the increased risk of suicide with *T. gondii* infection fits well into the theories of Stress-diathesis (9–12) and interpersonal theory of suicidal behavior (13), addressing chronic Toxoplasmosis to reduce the risk of suicide coherently aligns with the Social-Ecological Framework of Suicide Prevention (369). This framework is grounded in the Centers for Disease Control and Prevention framework for addressing health issues. Adopting a multi-level public health approach, Cramer and Kapusta (369), assert that suicide risk is associated with societal (e.g., poverty), community (e.g., barriers to health care access), relational (e.g., social isolation), and individual (e.g., chronic condition) influences. Conceptualizing suicide risk in this manner allows for multi-level interventions. Specifically, *T. gondii*, low-grade inflammation, and downstream molecular changes (e.g., low tyrosine and tryptophan, high kynurenine pathway metabolites) can be addressed at the level of socioeconomical predispositions (poverty and poor hygiene contributes to high seroprevalence), as preventative targets (e.g., food preparation and general hygiene) or as targetable biological mediators or moderators. This may ultimately contribute to a reduced burden of suicidal behavior and untimely mortality. In sum, our findings substantiate the need for longitudinal studies based on infection (seroconversion) and reactivation with monitoring of subsequent impulsivity, aggression, hopelessness/dysphoria, deficits in decisions making, and in the long run, randomized interventional experimental, preventative and risk lowering paradigms.

## Author Contributions

TP drafted the initial plan and the first draft of the manuscript that was reviewed and edited in detail by the other co-authors, provided the overall resubmission planning and pacing, and wrote the component on decision-making and *T. gondii*, as well as response to the reviewers. LB, DR, CL, and AH discussed the overall plan and alternatives. AW provided critical intellectual input into studies of *T. gondii* in individuals with mood disorders, contributed to the integration of text, figures, and legends, and modified figures. OO and DR provided critical intellectual input into suicide in schizophrenia. LB provided expertise in neuropsychological factors related to suicidal behavior, co-wrote the component on decision-making and suicide, and on the perspective of the Social-Ecological Framework of Suicide Prevention. CL provided a perspective of more general microbiome-gut-brain axis interactions. AH provided a perspective on the state-trait microbiome-gut-brain axis interactions. AD provided a perspective on the Amish specific exposure to *T. gondii* risk factors and clinical correlates and contributed to the integration of feedback from all co-authors. Upon resubmission, EB-G joined the team and provided substantial expertise on decision-making as a mediating mechanism and global perspectives on risk factors of suicide. All authors provided critical editing and intellectual input and approved the final version of the resubmitted manuscript.

## Conflict of Interest

The authors declare that the research was conducted in the absence of any commercial or financial relationships that could be construed as a potential conflict of interest.

## Glossary

AHR, aryl hydrocarbon receptor; ANCOVA, analysis of covariance; BH4, (6R)-L-erythro-5,6,7,8-tetrahydrobiopterin; CI, confidence interval; CMV, cytomegalovirus; CNS, central nervous system; CSF, cerebrospinal fluid; DALY, disability-adjusted life years; DSM-IV, *Diagnostic and Statistical Manual of Mental Disorders*, fourth edition; ELISA, enzyme-linked immunosorbent assay; FAF, The Questionnaire for Measuring Factors of Aggression (FAF-Fragebogen zur Erfassung von Aggressivitätsfaktoren); fMRI, functional magnetic resonance imaging; HPLC, high performance liquid chromatography; HSV1, herpes simplex virus 1; IBD, inflammatory bowel disease; IDO, indoleamine 2,3-dioxygenase; IED, Intermittent Explosive Disorder; IFN-γ, interferon gamma; IgG, immunoglobulin G; IgM, immunoglobulin M; IL, interleukin; ISI, Institute for Scientific Information; KYN, kynurenine; KYNA, kynurenic acid; LC, locus coeruleus; L-DOPA, l-3,4-dihydroxyphenylalanine; MCC, midcingulate cortex; NF-SSDV, non-fatal suicidal self-directed violence; NMDA, N-methyl-D-aspartate; NOS, Newcastle and Ottawa statements; OOA, Old Order Amish; OR odds ratio; PAF, population attributable fraction; PAH, phenylalanine hydroxylase; PANSS, Positive and Negative Syndrome Scale for Schizophrenia; Pglob, global preference; PHQ-2, Patient Health Questionnaire-2; PHQ-9, Patient Health Questionnaire-9; PIC, picolinic acid; PRISMA, Preferred Reporting Items for Systematic Reviews and Meta-Analyses; PROSPERO, The International Prospective Register of Systematic Reviews; QUIN, quinolinic acid; RR, risk ratio; SCID, Structured Clinical Interview for DSM Disorders; sgACC, subgenual anterior cingulate cortex; SSDV, suicidal self-directed violence; SSS-V, Sensation-Seeking Scale-V; SSS-V (DIS), Disinhibition subscale of the Sensation Seeking Scale-V; STROBE, Strengthening the Reporting of Observational Studies in Epidemiology; TBI, traumatic brain injury; TDO, tryptophan 2,3-dioxygenase; *T. gondii, Toxoplasma gondii*; Th1, T helper 1; TNF, tumor necrosis factor; Treg, regulatory T cell; TRP, tryptophan; US, United States; USA, United Stated of America; VMAT, vesicular monoamine transporter.
